# Dual Phosphorylation of STAT1 at Y701/S727 by TNFα Drives AIM2-Mediated PANoptosis of Renal Tubular Epithelial Cells and Fibrotic Progression in Renal Allografts

**DOI:** 10.7150/ijbs.123441

**Published:** 2026-01-01

**Authors:** Qianguang Han, Jiawen Liu, Jianjian Zhang, Qinghuan Shen, Junqi Zhang, Shuang Fei, Hao Chen, Li Sun, Zhengkai Huang, Zhijian Han, Jun Tao, Min Gu, Xiaobing Ju, Ruoyun Tan

**Affiliations:** 1 Department of Urology, the First Affiliated Hospital with Nanjing Medical University, Nanjing, 210029, China.; 2 Department of Urology, the Second Affiliated Hospital of Nanjing Medical University, Nanjing, 210028, China.

**Keywords:** chronic renal graft dysfunction (CAD), PANoptosis, TNF-α, AIM2, STAT1

## Abstract

Renal allograft interstitial fibrosis, a key pathological feature of chronic renal allograft dysfunction (CAD), is a critical determinant of long-term graft survival. However, its underlying molecular mechanisms remain incompletely understood. This study uncovers the central role of programmed cell death, particularly the novel PANoptosis modality, in the progression of CAD. PANoptosis integrates features of pyroptosis, apoptosis, and necroptosis, but does not fit within the confines of any single pathway, with its mechanisms previously undefined. By analyzing cell death patterns in CAD tissues through single-cell sequencing and validating findings via *in vivo* and *in vitro* experiments, this work demonstrates that in the context of chronic inflammation, tumor necrosis factor-alpha (TNF-α) modulates signal transducer and activator of transcription 1 (STAT1) through dual phosphorylation. This process directly induces tyrosine 701 phosphorylation and activates serine 727 phosphorylation via the p38 MAPK pathway. Phosphorylated STAT1 subsequently upregulates the PANoptosome sensor absent in melanoma 2 (AIM2), driving PANoptosis in renal tubular epithelial cells. This mechanism further exacerbates interstitial fibrosis by promoting the paracrine secretion of interleukin-6 and transforming growth factor-beta, which induces epithelial-mesenchymal transition (EMT) in adjacent tubular cells. These findings represent the first demonstration of the TNF-α/STAT1/AIM2 axis in triggering PANoptosis and its downstream EMT-fibrosis cascade, offering novel therapeutic targets for CAD intervention.

## Introduction

Renal transplantation is a critical renal replacement therapy that significantly improves the quality of life for patients with end-stage renal disease 1, 2. Chronic renal allograft dysfunction (CAD), the leading cause of graft failure, is pathologically characterized by abnormal extracellular matrix (ECM) deposition 3. Myofibroblasts, key effector cells in ECM synthesis and secretion, are closely associated with the severity of allograft interstitial fibrosis 4, 5. Despite this, the precise mechanisms driving allograft fibrogenesis remain poorly understood. Since renal tubular epithelial cells (RTECs) comprise 65% of renal parenchymal cells 6, 7, their injury is a central factor in fibrosis development, both by directly promoting interstitial fibrotic progression and by secreting proinflammatory and profibrotic factors that facilitate the transdifferentiation of neighboring cells into myofibroblasts 8, thereby perpetuating a fibrotic cycle.

Programmed cell death (PCD) encompasses various modalities, including apoptosis, necroptosis, pyroptosis, ferroptosis, and autophagy, all of which play essential roles in development, tissue remodeling, homeostasis, and disease pathogenesis 9. Although traditionally regarded as distinct pathways, emerging evidence highlights substantial crosstalk between pyroptosis, apoptosis, and necroptosis, leading to the identification of a novel inflammatory cell death process known as "PANoptosis"^10^, ^11^. This pathway integrates core features of all three PCD types but resists classification into any single category 12. PANoptosis regulates vital physiological processes such as immune defense, tissue repair, and cellular homeostasis and is implicated in various pathologies, including infections, metabolic disorders, immune-mediated diseases, and cancer 13, 14. Mechanistically, PANoptosis is driven by sensor proteins (e.g., ZBP1, AIM2, RIPK1, and NLRP12) 15, 16 which initiate signaling cascades that lead to the formation of the PANoptosome, a multiprotein complex that serves as a platform for activating the three PCD pathways and as a central signaling hub. These sensors are selectively activated by distinct stimuli. Although research on PANoptosis in renal allograft interstitial fibrosis remains sparse, its involvement in acute and chronic kidney injury 17, 18 and renal tumors 19, 20 is well-documented. Given the established roles of pyroptosis 21, apoptosis 22, and necroptosis 23 in renal transplantation, PANoptosis likely functions as an integrated cell death executor driving the progression of interstitial fibrosis in renal allografts.

As an emerging PCD modality that integrates the core features of pyroptosis, apoptosis, and necroptosis, PANoptosis offers a compelling focus for investigating the pathogenesis of CAD, given the established roles of individual PCD pathways in CAD progression. By analyzing clinical CAD specimens and utilizing in vivo and in vitro models, this study elucidates the critical role of PANoptosis in tubular epithelial cell injury and transdifferentiation during renal allograft interstitial fibrogenesis. Additionally, the mechanisms driving TNF-α-induced PANoptosis under chronic inflammatory conditions are explored, revealing its contribution to fibrosis progression and highlighting novel therapeutic targets for CAD intervention.

## Materials and Methods

### Patients and tissue samples

Renal transplant specimens were obtained from ten patients with a confirmed diagnosis of CAD, all of whom exhibited allograft interstitial fibrosis upon histopathological examination at Jiangsu Province Hospital. Normal renal tissue samples were sourced from nephrectomy specimens of patients with renal cell carcinoma (RCC), specifically excised from regions at least 5 cm from the tumor margin. The study protocol was approved by the Institutional Ethics Committee of Jiangsu Province Hospital and the Committee of the Second Affiliated Hospital of Nanjing Medical University (Ethical codes: 2022-KY-116-01; 2015-SRFA-096), and conducted in accordance with the Declaration of Helsinki. Informed consent was obtained from all participants.

### Animal treatment

C57BL/6 and BALB/c mice were obtained from the Laboratory Animal Center of Nanjing Medical University, with ethical approval granted by the University's Animal Research Ethics Committee (Ethical code: IACUC-1805014-2). A chronic allograft rejection model (Allo group) was established by orthotopic transplantation of kidneys from C57BL/6 donors into BALB/c recipients. Syngeneic controls (Syn group) were generated by transplanting kidneys from BALB/c donors into BALB/c recipients (**Figure S1A-C**). The chronic allograft fibrosis model was developed as previously described 24; with detailed procedural information provided in **Supplementary Materials 1**.

### Single-cell RNA sequencing (scRNA-seq)

Mice renal allograft tissues were processed into single-cell suspensions through enzymatic digestion. scRNA-seq libraries were prepared using the 10x Genomics Chromium Single Cell 3' Kit and sequenced on an Illumina NovaSeq platform. Raw sequencing data were processed with Cell Ranger for sample demultiplexing, barcode processing, and gene counting. Downstream analysis, including quality control, normalization, dimensionality reduction, and cell clustering, was performed using the Seurat R package. Cell types were annotated based on the expression of canonical marker genes.

### Cell culture and treatment

HK-2 cells (catalog no. SCSP-511) were obtained from the Cell Bank of the Type Culture Collection, Chinese Academy of Sciences. Renal fibroblast NRK-49F cells were sourced from the National Biomedical Laboratory Cell Resource Bank (Beijing, China). Cells were cultured in Dulbecco's Modified Eagle Medium/Ham's F-12 (DMEM-F12) supplemented with 10% (v/v) fetal bovine serum (FBS; AusGeneX, Australia) and 1% (v/v) penicillin-streptomycin (Gibco, USA) at 37°C in a humidified 5% CO_2_ incubator. The human monocytic leukemia cell line THP-1 was cultured in RPMI-1640 medium supplemented with 10% (v/v) FBS, 1% (v/v) P/S, and 0.05 mM β-mercaptoethanol at 37°C in a humidified 5% CO₂ incubator. Prior to co-culture, THP-1 cells were induced to adhere using 100 ng/ml phorbol 12-myristate 13-acetate (MCE, HY-18739). HK-2 cells were treated with TNF-α (0-200 ng/ml) (Novoprotein, C008 or R&D Systems, 210-TA) for 0-48 hours. Following initial dose optimization, TNF-α was applied at 100 ng/ml for 24 hours in subsequent *in vitro* experiments unless otherwise specified. Prior to stimulation, HK-2 cells were serum-starved (0% serum) for 12-24 hours, after which TNF-α treatment was carried out in medium supplemented with 2% serum.

To evaluate the functional contribution of specific profibrotic mediators, neutralizing antibodies were employed in a co-culture assay. The following neutralizing antibodies were used: IL-6 neutralizing antibody (Proteintech, 69001-1-Ig, 20 ng/mL) and TGF-β1 neutralizing antibody (Proteintech, 69012-1-Ig, 2.5 µg/mL). Antibodies were added to the medium 30 minutes prior to co-culture with TNF-α-primed HK-2 cells, after which co-culture was conducted for an additional 48 hours.

### Lentivirus, plasmids, and reagents

Lentiviral constructs for AIM2 knockdown, AIM2 overexpression, STAT1 knockdown, and corresponding empty vectors were purchased from Genechem (Shanghai, China), with cell treatments following the manufacturer's protocols. Additionally, STAT1 phospho-site mutant plasmids (STAT1-Y701F [tyrosine→phenylalanine], STAT1-S727A [serine→alanine]) and the wild-type STAT1 plasmid were obtained from Genechem (Shanghai, China). Various inhibitors were sourced from MedChemExpress (MCE), including Ac-YVAD-cmk (Pyroptosis inhibitor, HY-16990), Z-VAD-FMK (Apoptosis inhibitor, HY-16658B), Necrosulfonamide (Necroptosis inhibitor, HY-100573), MG-132 (HY-13259), Chloroquine (CQ, HY-17589A), Fludarabine (STAT1 inhibitor, HY-B0069), MK-2206 (AKT inhibitor, HY-108232), QNZ (NF-κB inhibitor, HY-13812), SB203580 (p38MAPK inhibitor, HY-10256), SP600125 (JNK inhibitor, HY-12041), U0126 (MEK/ERK inhibitor, HY-12031A), and R7050 (TNF-α Antagonist, HY-110203).

### RNA isolation and reverse-transcriptase qPCR (RT-qPCR) assay

Total RNA was extracted from renal tissues and cell lines using TRIzol reagent (Invitrogen, 15596026) following the manufacturer's instructions. RNA was reverse-transcribed using HiScript® III All-in-one RT SuperMix (Vazyme, R333-01). Quantitative PCR (qPCR) was performed using ChamQ SYBR Color qPCR Master Mix (Vazyme, Q431-02), with each reaction conducted in triplicate. Primer sequences are listed in Supplementary Data (**Table S1**).

### Western blot assay

Total protein was extracted using RIPA lysis buffer (Beyotime, Shanghai), and protein concentrations were quantified using the BCA assay. For Western blot, proteins were separated via SDS-PAGE and transferred to polyvinylidene fluoride (PVDF) membranes. Membranes were incubated with primary antibodies (**Table S2**) overnight at 4°C, followed by incubation with secondary antibodies (anti-mouse and anti-rabbit) from Proteintech at a dilution of 1:5000 (SA00001-1, SA00001-2).

### Chromatin immunoprecipitation (ChIP) assay

ChIP assays were conducted using the ChIP-IT Express Kit (Active Motif, 53008) per the manufacturer's protocol. Genomic DNA was sheared into fragments ranging from 300 to 1000 bp. Equal amounts of DNA were incubated overnight at 4°C with either phospho-STAT1 (p-STAT1) antibody (CST, 9167) or IgG-negative control antibody (CST, 2729), along with 20 μL magnetic protein G beads. After decrosslinking and proteinase treatment, immunoprecipitated DNA was purified and analyzed by standard PCR. The promoter region of AIM2 and corresponding primer sequences for binding sites are provided in Supplementary Data (**Table S3**).

### Dual luciferase reporter assay

The wild-type AIM2 promoter fragment (-1999 to 0) was cloned into the pGL3-basic vector to generate the pGL3-AIM2-WT reporter plasmid. Using this as a template, two mutant plasmids, pGL3-AIM2-MUT1 and pGL3-AIM2-MUT2, were constructed by introducing specific mutations into the STAT1-binding elements at positions -1434 to -1442 and -900 to -908, respectively. HK-2 cells were co-transfected with either the WT or mutant reporter plasmids (pGL3-based constructs) and the pRL-TK plasmid encoding Renilla luciferase as an internal control. After transfection, cells were treated with TNF-α or left untreated. At 24-48 hours post-transfection, cells were harvested, and luciferase activity was measured using a dual-luciferase reporter assay kit (Vazyme, DL101-01) following the manufacturer's protocol. Firefly luciferase activity (driven by the AIM2 promoter constructs) and Renilla luciferase activity (driven by pRL-TK) were quantified sequentially, with Firefly luciferase activity normalized to corresponding Renilla luciferase activity for each sample.

### Histopathological analysis

After fixation in paraformaldehyde, kidneys were embedded in paraffin, and serial sections of 4 μm thickness were cut using a microtome. Sections were stained with H&E, Masson's trichrome, and Periodic Acid-Schiff (PAS) for histopathological analysis as previously described 25.

### Immunohistochemistry (IHC) and Immunofluorescence (IF)

Tissue sections were deparaffinized and subjected to antigen retrieval to unmask epitopes. Following the blocking of non-specific binding sites, sections were incubated with the appropriate primary antibodies (**Table S2**). For immunohistochemistry (IHC), secondary antibodies from the IHC kit (Absin, abs996) were applied at room temperature for 30 minutes. Chromogenic detection was performed using Diaminobenzidine (DAB), followed by counterstaining with hematoxylin. Multiplex immunofluorescence was conducted using a commercial kit (Absin, abs50015) according to the manufacturer's protocol. Primary antibodies for immunofluorescence are listed in **Table S2**. Secondary antibodies included: Alexa Fluor 488-Conjugated Anti-Rabbit IgG (H+L) (CST, 4412S, 1:500) and Alexa Fluor 594-Conjugated Anti-Mouse IgG (H+L) (CST, 8890S, 1:500). Nuclei were counterstained with 4',6-diamidino-2-phenylindole (DAPI), and slides were coverslipped for imaging.

### Flow cytometry and cell viability assay

Cells were trypsinized, harvested, and adjusted to a density of 1 × 10⁶ cells/mL. The cell pellet was resuspended in 100 μL of binding buffer and stained with cleaved-GSDMD (CST, 36425, 1:100), anti-cleavedCASP3 (CST, 9603, 1:100), and anti-pMLKL (Abcam, ab187091, 1:50). After centrifugation, the pellet was stained with anti-rabbit Alexa Fluor 488 secondary antibody (Invitrogen, #A21206). Following incubation, 10 μL of propidium iodide (PI) solution (50 μg/mL) was added, and cells were incubated for 5 minutes on ice. This protocol identifies cells with compromised membrane integrity through PI uptake (detected by red fluorescence). Cell suspensions were analyzed using a CytoFLEX flow cytometer (Beckman Coulter), and data analysis was performed with CytExpert software (Beckman Coulter). Cell viability was assessed using the Calcein/PI Cell Viability/Cytotoxicity Assay Kit (Beyotime, C2015S) according to the manufacturer's instructions.

### Enzyme-Linked Immunosorbnent Assay (ELISA)

The levels of Interleukin-1β (IL-1β), Transforming Growth Factor-β1 (TGF-β1), Platelet-Derived Growth Factor-BB (PDGF-BB), and Interleukin-6 (IL-6) in the cell culture medium were measured using ELISA kits for IL-1β (mlbio, China, YJ058059), TGF-β1 (mlbio, China, YJ022522), PDGF-BB (mlbio, China, YJ023009), and IL-6 (mlbio, China, YJ028583), following the manufacturer's instructions.

### Data collection and statistical analysis

The GSE9493, GSE76882, and GSE21374 datasets were retrieved from the Gene Expression Omnibus (GEO) database (https://www.ncbi.nlm.nih.gov/geo/). The GSE9493 dataset included 25 cases of "chronic allograft nephropathy (CAN)" and 15 control samples from nephrectomy patients. The GSE76882 dataset comprised an "Interstitial Fibrosis and Tubular Atrophy (IFTA)" group and 99 cases in the "Normal Functional Transplants" group. The GSE21374 dataset contained 282 kidney biopsy specimens collected post-transplantation. Additionally, RNA sequencing was performed on HK-2 cells treated with TNF-α (100 ng/mL) for 24 hours (3 groups) and control cells (3 groups). Differential gene expression analysis was conducted using the “limma” package in R. Gene Ontology (GO) and Kyoto Encyclopedia of Genes and Genomes (KEGG) enrichment analyses were also performed using R. Survival analysis, incorporating graft survival time, was conducted with the “survival” package. Statistical analyses were performed using GraphPad Prism 8.3.0, with data presented as mean ± standard error of the mean (SEM). Student's t-test was used for comparisons between two groups, while one-way or two-way ANOVA with Tukey's or Dunnett's post hoc tests was applied for multiple group comparisons. Statistical significance was defined as follows: *P < 0.05, **P < 0.01, ***P < 0.001; ns, not significant.

## Results

### Activation of pyroptosis, apoptosis, and necroptosis pathways in RTECs in human CAD allografts

**Figure 1:**
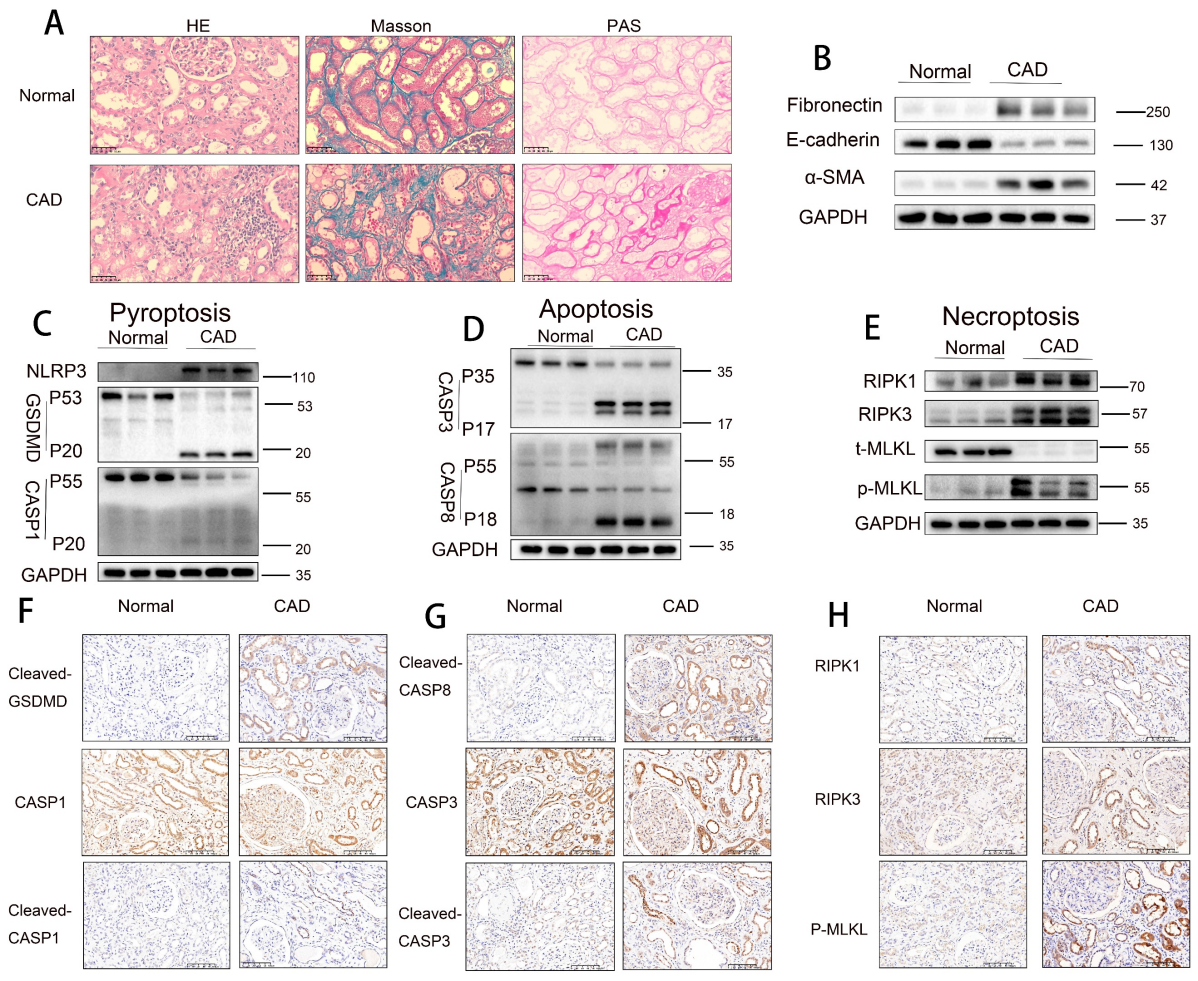
** Activation of pyroptosis, apoptosis, and necroptosis pathways in RTECs in human CAD allografts. A**) Representative H&E, Masson's trichrome, and PAS staining of renal tissue from normal controls and CAD patients. **B**) Western blot analysis of fibronectin, E-cadherin, and α-SMA protein levels in renal tissue from normal controls and CAD patients. **C-E**) Western blot analysis of: (**C**) NLRP3, full-length/cleaved GSDMD, and full-length/cleaved CASP1 (p55/p20); (**D**) full-length/cleaved CASP3 (p35/p17) and full-length/cleaved CASP8 (p55/p18); (**E**) RIPK1, RIPK3, total MLKL (t-MLKL), and phosphorylated MLKL (p-MLKL) protein levels in renal tissue from normal controls and CAD patients. **F-H**) Representative immunohistochemistry (IHC) images (200× magnification) showing: (**F**) cleaved-GSDMD and full-length/cleaved CASP1 (p55/p20); (**G**) cleaved CASP8 (p18) and full-length/cleaved CASP3 (p35/p17); (**H**) RIPK1, RIPK3, and p-MLKL expression in renal tissue from normal controls and CAD patients.

Histopathological staining (H&E, Masson trichrome, and PAS) revealed that renal allograft tissues from the CAD group exhibited significantly more severe tubular atrophy, tissue injury, interstitial fibrosis, and glycogen deposition compared to normal control tissues (**Figure 1A**). Western blot analysis demonstrated a marked downregulation of E-cadherin expression and significant upregulation of α-smooth muscle actin (α-SMA) and fibronectin levels in CAD renal tissues relative to controls (**Figure 1B**), indicating the presence of epithelial-mesenchymal transition (EMT) and interstitial fibrosis in CAD. Further immunoblotting analysis revealed notably elevated levels of pyroptosis-related markers (NLRP3, cleaved GSDMD, and cleaved CASP1), apoptosis-related markers (cleaved CASP3 [p17] and cleaved CASP8 [p18]), and necroptosis-related markers (RIPK1, RIPK3, and phosphorylated MLKL [p-MLKL]) in CAD renal tissues compared to controls (**Figure 1C-E**). Immunohistochemical staining confirmed the significantly increased expression of pyroptosis markers cleaved GSDMD and cleaved CASP1 (p20), apoptosis markers cleaved CASP3 (p17) and cleaved CASP8 (p18), and necroptosis marker p-MLKL in CAD renal tissues, with predominant localization in RTECs (**Figure 1F-H**). Immunofluorescence colocalization analysis showed positive colocalization of the pyroptosis marker cleaved CASP1, apoptosis marker cleaved CASP3, necroptosis marker p-MLKL, and the proximal tubule marker AQP1 (**Figure S2A-C**). Collectively, these results indicate significant interstitial fibrosis in CAD renal allografts, alongside marked activation of pyroptotic, apoptotic, and necroptotic pathways, specifically within proximal RTECs.

### Pyroptosis, apoptosis, and necroptosis are activated in RTECs of allografts in a murine model of CAD

**Figure 2:**
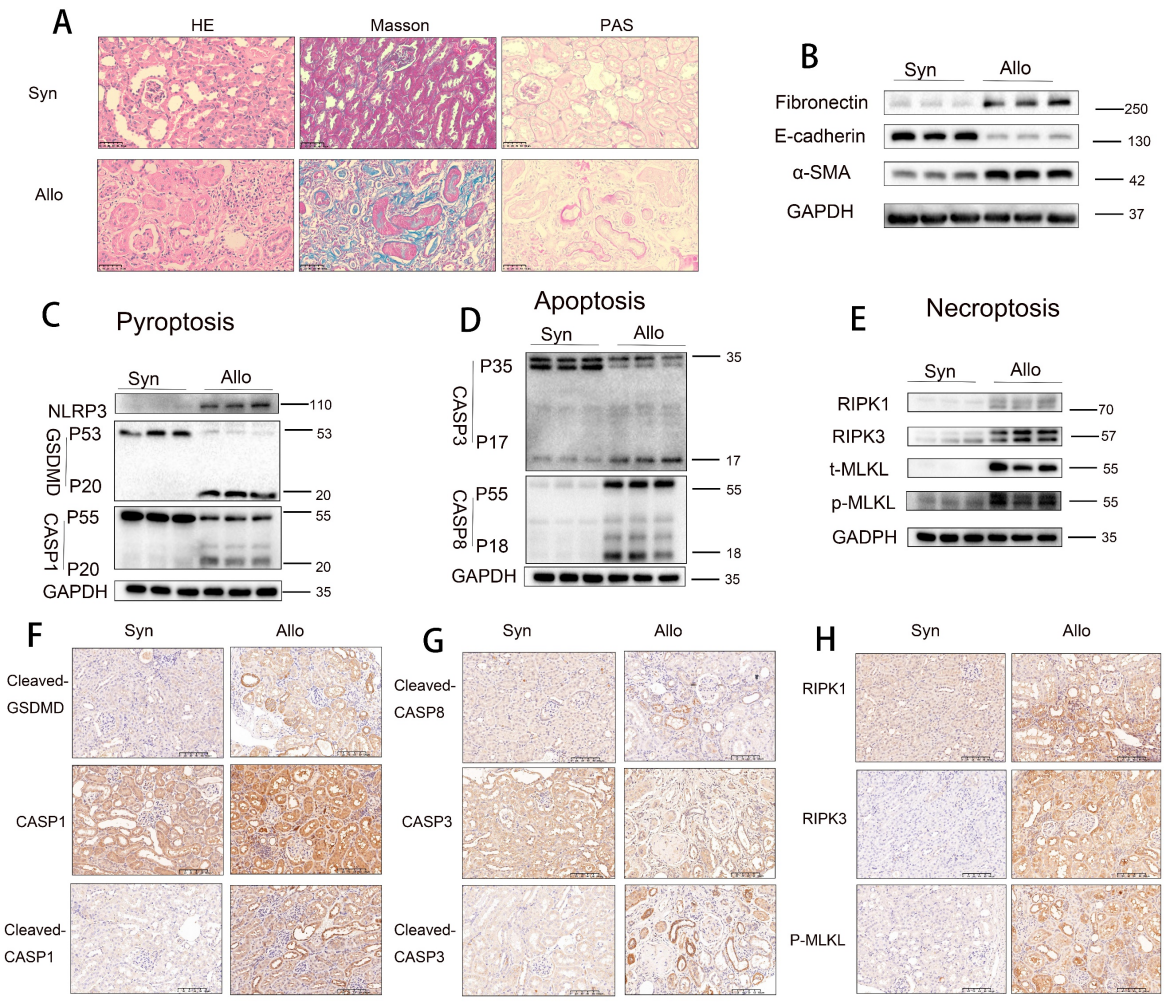
** Pyroptosis, apoptosis, and necroptosis activation in RTECs of allografts in a murine model of CAD. A)** Representative H&E, Masson's trichrome, and PAS staining of renal tissue from Syn and Allo groups. **B**) Western blot analysis of fibronectin, E-cadherin, and α-SMA protein levels in renal tissue from Syn and Allo groups. **C-E**) Western blot analysis of: (**C**) NLRP3, full-length/cleaved GSDMD and full-length/cleaved CASP1 (p55/p20); (**D**) full-length/cleaved CASP3 (p35/p17) and full-length/cleaved CASP8 (p55/p18); (**E**) RIPK1, RIPK3, total MLKL (t-MLKL), and phosphorylated MLKL (p-MLKL) protein levels in renal tissue from Syn and Allo groups. **F-H**) Representative immunohistochemistry (IHC) images depicting, 200×, (**F**) Cleaved-GSDMD and full-length /cleaved CASP1 (p55/p20); (**G**) cleaved CASP8 (p18) and full-length /cleaved CASP3 (p35/p17); (**H**) RIPK1, RIPK3 and p-MLKL expression in renal tissue from Syn and Allo groups.

To validate these observations, further experiments were conducted using an animal model. Histopathological staining (H&E, Masson trichrome, and PAS) revealed that renal tissues from the Allo group exhibited more severe tubular atrophy, tissue injury, interstitial fibrosis, and glycogen deposition compared to those from the Syn group (**Figure 2A**). Western blot analysis demonstrated significant downregulation of E-cadherin and marked upregulation of α-SMA and fibronectin levels in Allo renal tissues (**Figure 2B**). Additionally, markers of pyroptosis (NLRP3, cleaved GSDMD, and cleaved CASP1), apoptosis (cleaved CASP3 [p17] and cleaved CASP8 [p18]), and necroptosis (RIPK1, RIPK3, and p-MLKL) were significantly elevated in the Allo group compared to the Syn group (**Figure 2C-E**). Immunohistochemical staining confirmed the markedly increased expression of the pyroptosis markers cleaved GSDMD and cleaved CASP1 (p20), apoptosis markers cleaved CASP3 (p17) and cleaved CASP8 (p18), and necroptosis marker p-MLKL in Allo renal tissues, predominantly in RTECs (**Figure 2F-H**), indicating a pathological consistency between the allograft model and clinical CAD tissues. scRNA-seq analysis revealed (i) enhanced activation of multiple PCD pathways, including pyroptosis, apoptosis, necroptosis, autophagy-related cell death, ferroptosis, cuproptosis, disulfidptosis, lysosome-dependent cell death, and zinc-dependent cell death in the Allo group relative to the Syn group (**Figure S3A**); (ii) dominant activation of apoptosis, pyroptosis, and necroptosis in RTECs (**Figure S3B**); and (iii) classification of these three PCD types as “PANoptosis,” with scRNA-seq confirming its predominant occurrence in RTECs (**Figure S3C**).

### Activation of “PANoptosis” in RTECs of renal tissue from human and murine CAD groups

**Figure 3:**
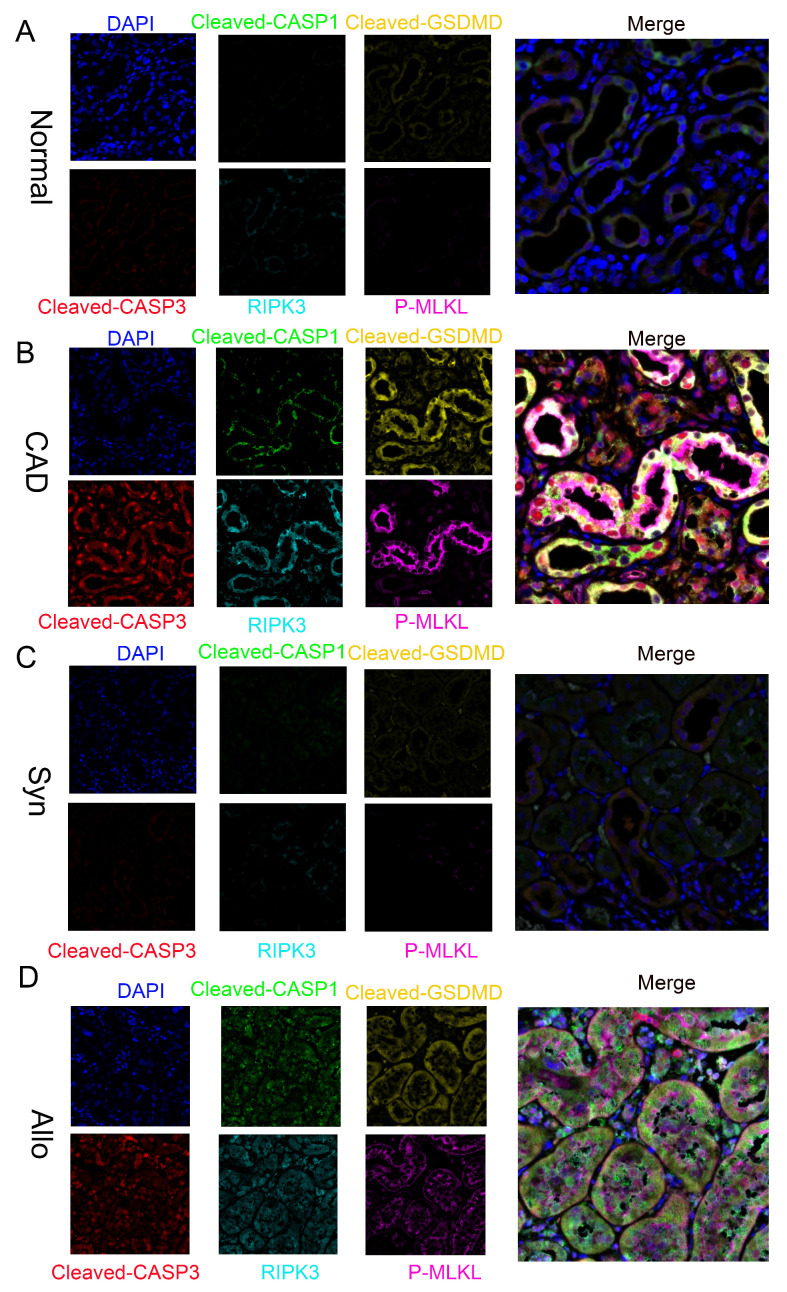
** Activation of PANoptosis in RTECs of human and murine renal allografts. A-D**) Representative multiplex fluorescence IHC images showing cleaved CASP1 (p20), cleaved GSDMD, cleaved CASP3 (p17), RIPK3, and p-MLKL in renal tissue from the normal, CAD, Syn, and Allo groups.

Further multiplex immunofluorescence analysis revealed significantly enhanced signals for pyroptosis markers cleaved CASP1 and cleaved GSDMD, the apoptosis marker cleaved CASP3, and necroptosis markers RIPK3 and p-MLKL in CAD renal tissues compared to normal controls. Notably, these markers colocalized within individual RTECs (**Figure 3A-B**). Similar results were observed in the Syn and Allo mouse models (**Figure 3C-D**), confirming the concurrent activation of pyroptosis, apoptosis, and necroptosis pathways—collectively termed "PANoptosis"—in RTECs of both CAD patients and renal allograft mice.

### TNF pathway activation during CAD progression

**Figure 4:**
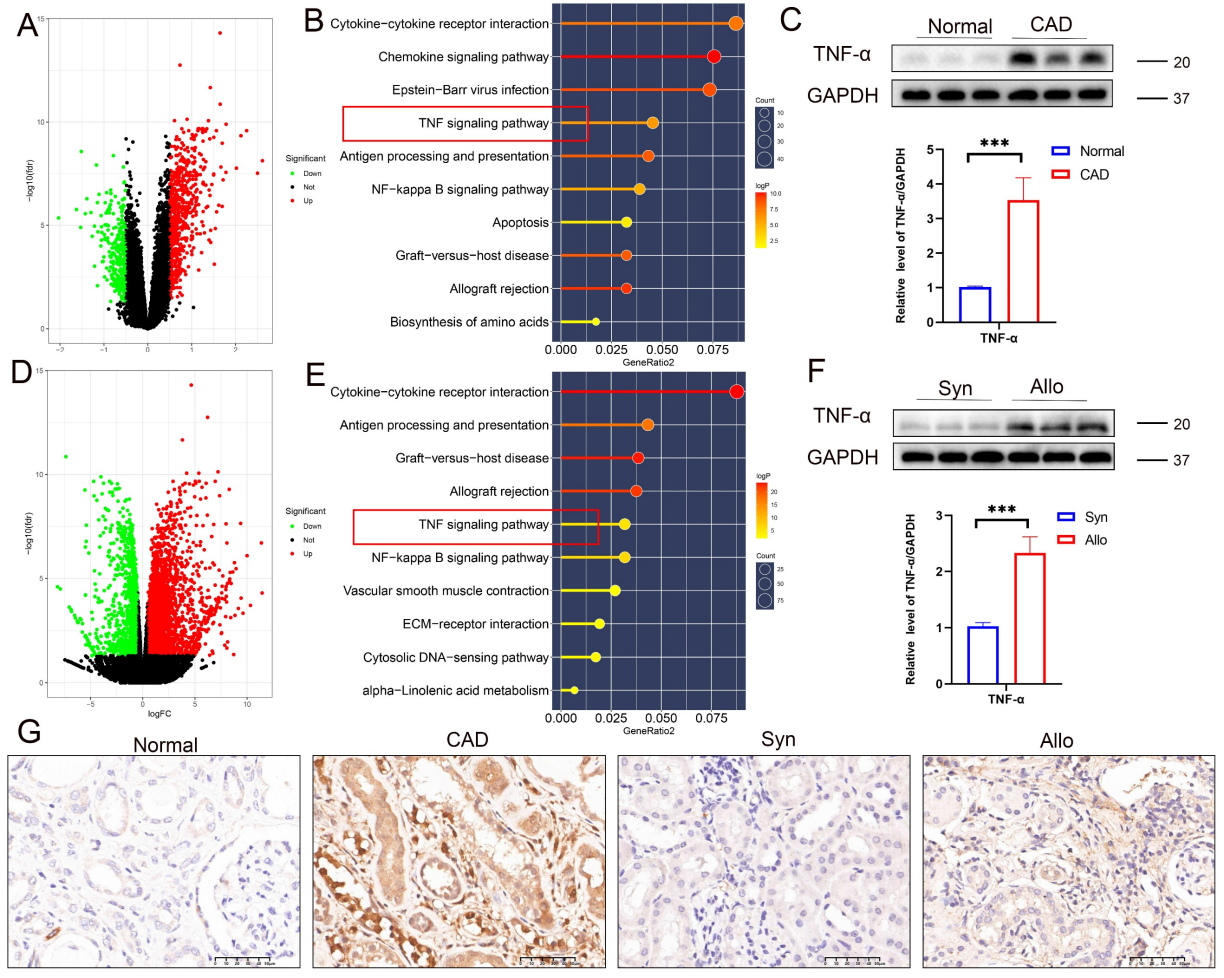
**TNF-α pathway activation during CAD progression. A)** Volcano plot of differentially expressed genes (DEGs) between normal and CAD groups in the GSE76882 dataset. **B**) KEGG pathway enrichment analysis of DEGs between normal and CAD groups. **C**) Western blot and quantification of TNF-α protein levels in renal tissue from normal and CAD groups. **D**) Volcano plot of DEGs between Syn and Allo groups. **E**) KEGG pathway enrichment analysis of DEGs between Syn and Allo groups. **F**) Western blot and quantification of TNF-α protein levels in renal tissue from Syn and Allo groups. **G**) Representative IHC images showing TNF-α expression in renal tissue from the normal, CAD, Syn, and Allo groups.

Analysis of the GSE76682 dataset identified 916 differentially expressed genes (DEGs), including 618 upregulated and 298 downregulated genes (volcano plot, **Figure 4A**). KEGG pathway enrichment analysis of DEGs highlighted significant enrichment in the TNF signaling pathway (**Figure 4B**), suggesting that TNF plays a critical role in renal allograft interstitial fibrosis, consistent with our previous findings 24-26. Western blot analysis confirmed significantly elevated TNF-α protein levels in CAD renal tissues compared to controls (**Figure 4C**). Transcriptomic sequencing of renal allografts from Syn and Allo mouse models identified 2226 DEGs (1713 upregulated and 513 downregulated; volcano plot, **Figure 4D**), with KEGG analysis also showing significant enrichment in the TNF signaling pathway (**Figure 4E**). Western blot analysis confirmed markedly higher TNF-α expression in the Allo group compared to the Syn group (**Figure 4F**). Immunohistochemical validation further demonstrated significantly increased TNF-α expression in the renal interstitium and tubules of CAD tissues compared to normal kidneys, with pronounced upregulation of TNF-α in the renal interstitium and tubules of the Allo group relative to Syn controls (**Figure 4G**).

### TNF-α could induce PANoptosis in RTECs

**Figure 5:**
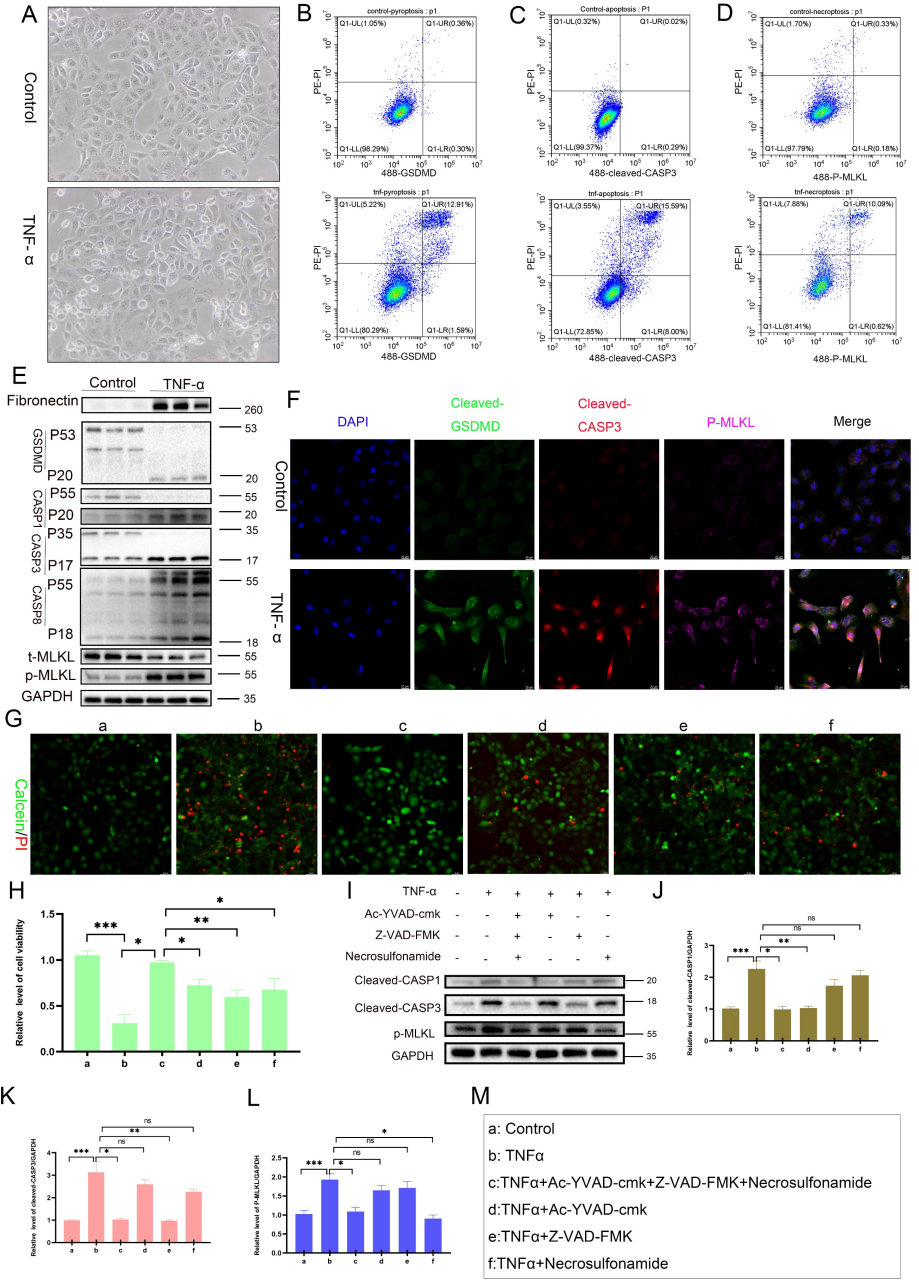
** TNF-α induces PANoptosis in RTECs. A)** Representative light-microscopy images of HK-2 cells from the control group or TNF-α (100 ng/mL) group. **B**) Flow-cytometric quantification of the percentage of cleaved-GSDMD^+^ PI^+^ HK-2 cells from the control group or TNF-α (100 ng/mL) group. **C**) Flow-cytometric quantification of the percentage of cleaved CASP3^+^ PI^+^ in HK-2 cells from the control group or TNF-α (100 ng/mL) group. **D**) Flow-cytometric quantification of the percentage of p-MLKL^+^ PI^+^ in HK-2 cells from the control group or TNF-α (100 ng/mL) group. **E**) Western blot analysis of the protein levels of Fibronectin, full-length/cleaved GSDMD, CASP1 (p55 and p20), full-length/cleaved CASP3, full-length/cleaved CASP8, t-MLKL, and p-MLKL in HK-2 cells from the control group or TNF-α (100 ng/mL) group. **F**) Representative IF images of cleaved GSDMD, cleaved CASP3 (p17), and p-MLKL expression in HK-2 cells from the control group and TNF-α (100 ng/mL) group. **G**) Representative IF images of Calcein-AM/PI in HK-2 cells treated with TNF-α supplemented with or without candidates (Ac-YVAD-cmk [20 µM], Z-VAD-FMK [20 µM], or Necrosulfonamide [5 µM]) for 24 h. **H**) Quantitative analysis of cell viability, *P < 0.05, ** P < 0.01, *** P < 0.001. **I-L**) Western blot and quantitative analysis of cleaved CASP1 (p20), cleaved CASP3 (p17), and p-MLKL in HK-2 cells treated with TNF-α supplemented with or without candidates (Ac-YVAD-cmk, Z-VAD-FMK, or Necrosulfonamide) for 24 h. *P < 0.05, ** P < 0.01, *** P < 0.001. **M**) Labels of cell treatment conditions: (a) Control, (b) TNF-α, (c) TNF-α+Ac-YVAD-cmk+Z-VAD-FMK+Necrosulfonamide, (d) TNF-α+Ac-YVAD-cmk, (e) TNF-α+Z-VAD-FMK, (f) TNF-α+Necrosulfonamide.

*In vitro* experiments demonstrated that TNF-α stimulation induced characteristic morphological changes in RTECs, including membrane blebbing and cell shrinkage (**Figure 5A**). Flow cytometry showed significantly increased proportions of PI-positive (membrane-disrupted) cells coexpressing pyroptosis marker cleaved GSDMD (**Figure 5B**), apoptosis marker cleaved CASP3 (p17) (**Figure 5C**), and necroptosis marker p-MLKL (**Figure 5D**) in TNF-α-treated cells. Western blot analysis confirmed marked upregulation of fibronectin, cleaved GSDMD, cleaved CASP1, cleaved CASP3 (p17), cleaved CASP8 (p18), and p-MLKL in TNF-α-exposed cells relative to controls (**Figure 5E**), with immunofluorescence validating coactivation (**Figure 5F**). These results indicate that TNF-α triggers the concurrent activation of pyroptosis, apoptosis, and necroptosis pathways, collectively activating PANoptosis in single RTECs. Calcein-AM/PI viability assays confirmed significant TNF-α-induced cytotoxicity, which could not be fully rescued by inhibiting pyroptosis, apoptosis, or necroptosis individually (**Figure 5G-H**). High-concentration inhibitors also failed to fully rescue TNF-α-induced cytotoxicity (**Figure S4A-F**). Western blot analysis revealed pathway-specific rescue effects: pyroptosis inhibition selectively abrogated TNF-α-induced cleaved CASP1 elevation (**Figure 5I, J**), apoptosis inhibition specifically suppressed cleaved CASP3 expression (**Figure 5I, K**), and necroptosis inhibition selectively reduced p-MLKL levels (**Figure 5I, L**). These results demonstrate that TNF-α activates PANoptosis in RTECs.

### TNF-α induces PANoptosis via upregulation of AIM2 in RTECs

**Figure 6:**
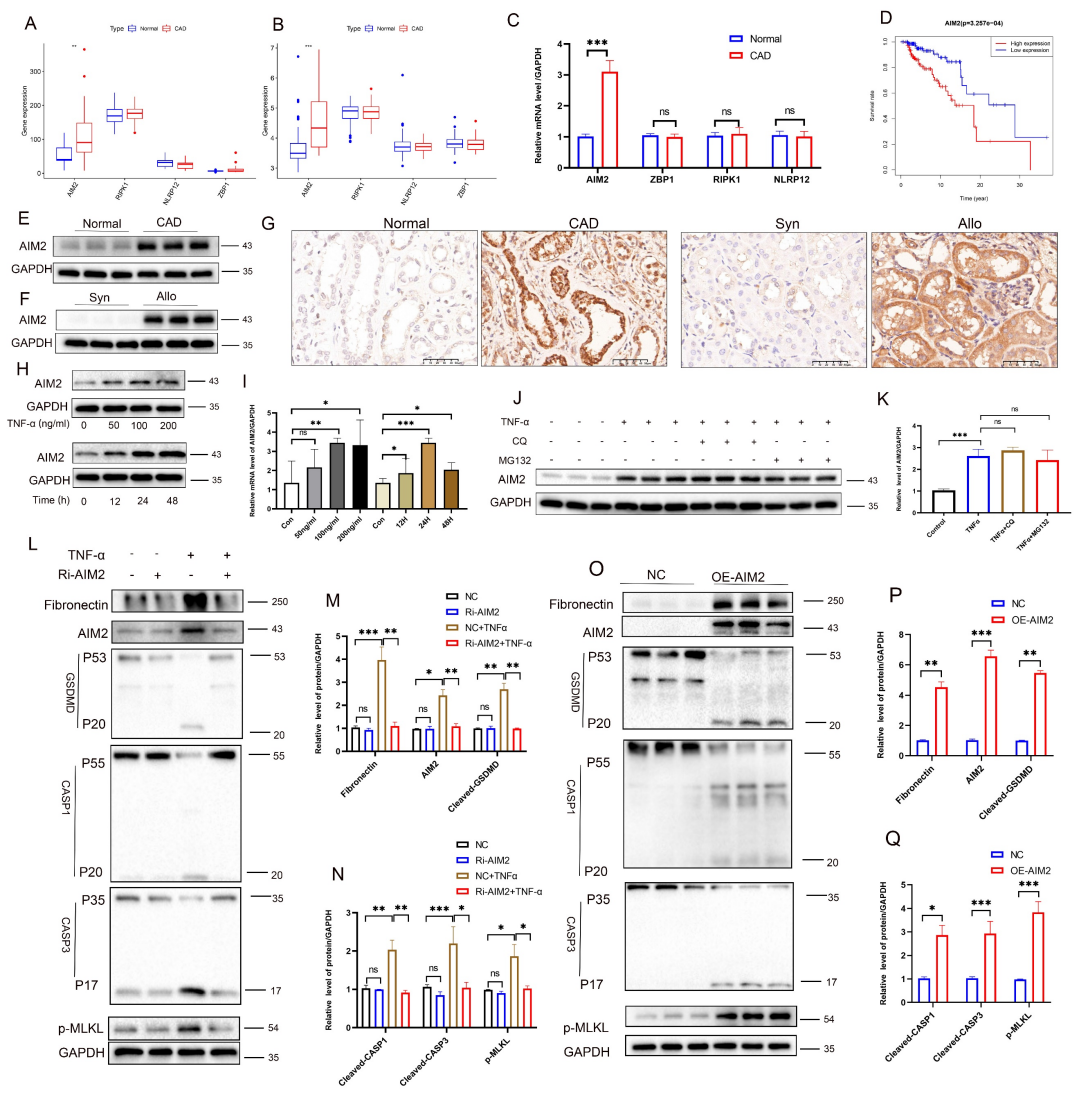
** TNF-α induces PANoptosis *via* upregulation of AIM2 in RTECs. A-B**) Boxplots of AIM2, ZBP1, RIPK1, and NLRP12 expression levels in the GSE9493 and GSE76882 datasets.** C**) qRT-PCR analysis of relative AIM2, ZBP1, RIPK1, and NLRP12 mRNA levels in normal and CAD groups. **D**) Kaplan-Meier curve for graft survival time stratified by AIM2 expression levels (red: high expression; blue: low expression). **E-F**) Western blot analysis of AIM2 protein levels in renal tissue from normal, CAD, Syn, and Allo groups. **G**) Representative IHC images of AIM2 expression in renal tissue from the normal, CAD, Syn, and Allo groups. **H**) Western blot analysis of AIM2 protein levels in HK-2 cells treated with TNF-α (0, 50, 100, and 200 ng/mL) for 24 h and treated with TNF-α (100 ng/mL) for 0, 12, 24, or 48 h. **I**) qRT-PCR analysis of relative mRNA levels of AIM2 in HK-2 cells treated with TNF-α (0, 50, 100, and 200 ng/mL) for 24 h and treated with TNF-α (100 ng/mL) for 0, 12, 24, or 48 h. **J-K**) Western blot and quantitative analysis of AIM2 in HK-2 cells treated with TNF-α supplemented with or without candidates (CQ or MG132) for 24 h. *P < 0.05, ** P < 0.01, *** P < 0.001. **L-N**) Western blot and quantitative analysis of Fibronectin, AIM2, full-length/cleaved GSDMD, full-length/cleaved CASP1, full-length/cleaved CASP3, and p-MLKL in HK-2 cells from the control groups (NC) and AIM2 knockdown groups (Ri-AIM2) treated with TNF-α (100 ng/mL, 24 h). **O-Q**) Western blot and quantitative analysis of Fibronectin, AIM2, full-length/cleaved GSDMD, full-length/cleaved CASP1, full-length/cleaved CASP3, and p-MLKL in HK-2 cells from the control groups (NC) and AIM2 overexpression groups (OE-AIM2). *P < 0.05, ** P < 0.01, *** P < 0.001.

Activation of PANoptosis requires specific sensor proteins. Analysis of the GSE9493 and GSE76882 datasets revealed that, among four candidate PANoptosis sensors (AIM2, ZBP1, RIPK1, and NLRP12), only AIM2 exhibited significantly upregulated expression in CAD renal tissues compared to controls (**Figure 6A-B**), a finding validated by PCR (**Figure 6C**). Both PCR and Western blot analysis showed that, upon TNF-α stimulation, only AIM2 was significantly increased among the four sensors (**Figure S5C-D**). Integration of the GSE21374 dataset with renal allograft survival data demonstrated a correlation between high AIM2 expression and shorter allograft survival, positioning AIM2 as an independent risk factor for graft failure (**Figure 6D**). Western blot analysis further confirmed markedly elevated AIM2 levels in CAD tissues compared to normal renal tissues (**Figure 6E**). Additionally, AIM2 levels were significantly higher in Allo murine kidneys compared to Syn murine kidneys (**Figure 6F**). IHC revealed specific enrichment of AIM2 in damaged tubules of CAD patients and Allo mice (**Figure 6G**). The results of scRNA-seq analysis indicate that AIM2 level elevated in TNF-α^+^ cell populations (**Figure S5E**). *In vitro*, TNF-α stimulation induced a time- and dose-dependent upregulation of AIM2 in RTECs, with peak expression observed at 100 ng/mL for 24 hours (**Figure 6H-I**, Western blot and PCR results). Notably, blocking protein degradation pathways using the autophagy inhibitor CQ or the proteasome inhibitor MG132 did not affect AIM2 levels (**Figure 6J-K**), a result consistent throughout the 0-48-hour TNF-α stimulation period on HK-2 cells (**Figure S5A-B**). Functional studies involving AIM2 knockdown and overexpression in HK-2 cells demonstrated that AIM2 knockdown significantly attenuated the TNF-α-induced upregulation of cleaved GSDMD, cleaved CASP1, cleaved CASP3, p-MLKL, and the fibrotic marker fibronectin (**Figure 6L-N**). In contrast, AIM2 overexpression directly activated these cell death pathways and fibrotic markers (**Figure 6O-Q**). Collectively, these results establish that TNF-α triggers PANoptosis in RTECs through the transcriptional upregulation of AIM2. Similar results were obtained upon stimulation of cells with TNF-α from R&D Systems (**Figure S6A-G**).

### Phosphorylated STAT1 modulates AIM2 expression and RTEC PANoptosis

**Figure 7:**
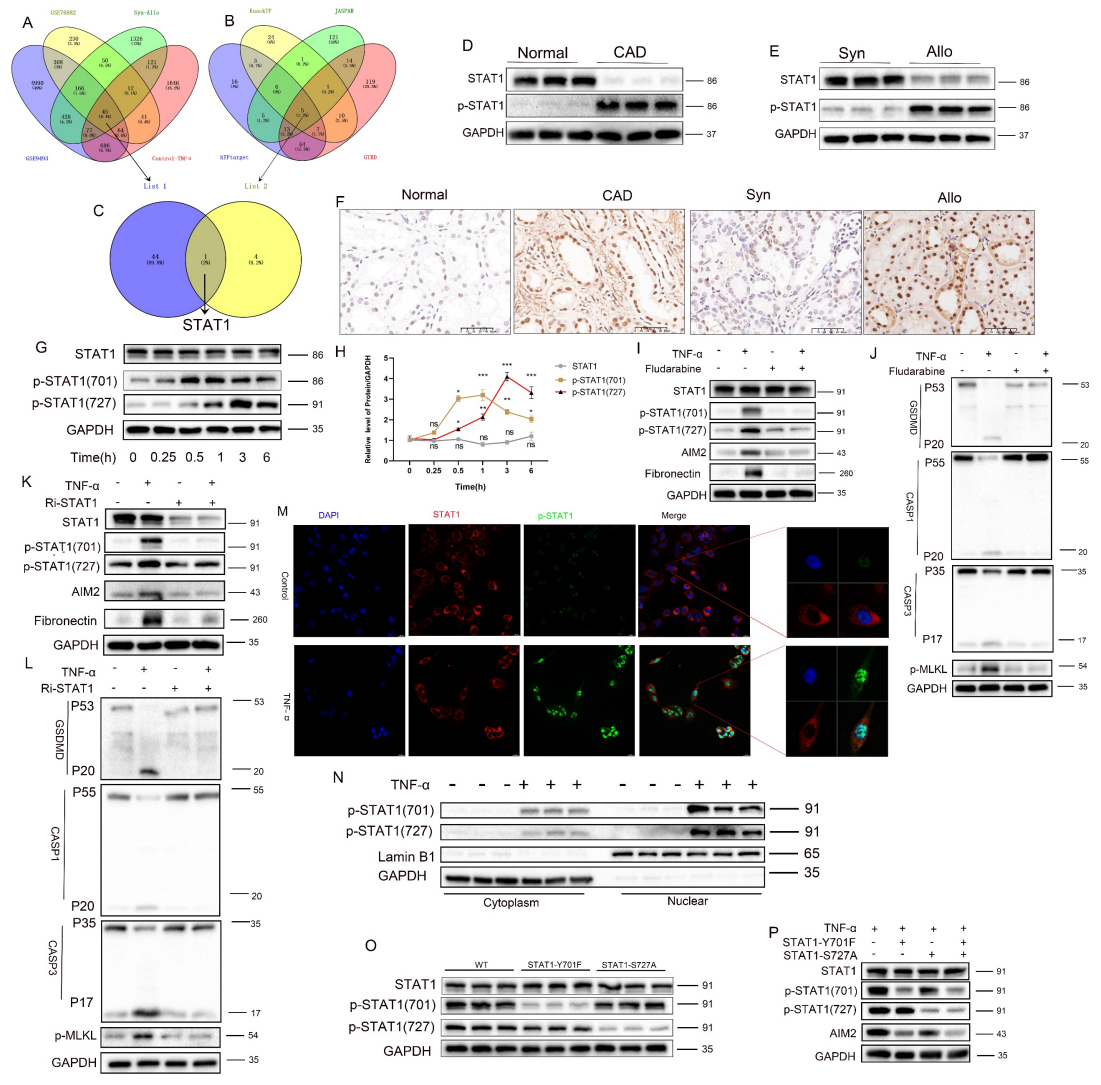
** Phosphorylated STAT1 modulates AIM2 expression and RTEC PANoptosis. A**) Venn diagram of differentially expressed genes (DEGs) among the GSE9493 and GSE76882 datasets, murine renal allograft RNA-seq (Syn vs Allo), and HK-2 cell RNA-seq (control vs TNF-α). **B**) Venn diagram of transcription factors whose putative binding sites within the AIM2 promoter were predicted by hTFtarget, KnockTF, JASPAR, and GTRD. **C**) Overlap between DEGs and predicted transcription factors. **D-E**) Western blot analysis of the protein levels of STAT1 and p-STAT1 in renal tissue from normal, CAD, Syn, and Allo groups. **F**) Representative IHC images of p-STAT1 expression in renal tissue from normal, CAD, Syn, and Allo groups. **G-H**) Western blot and quantitative analysis of the protein levels of STAT1, p-STAT1 (Tyr701), and p-STAT1 (Ser727) in HK-2 cells treated with TNF-α (100 ng/mL) for 0, 0.25, 0.5, 1, 3, or 6 h. **I-J**) Western blot analysis of the protein levels of (**I**) STAT1, p-STAT1 (Tyr701), p-STAT1 (Ser727), AIM2, and Fibronectin; (**J**) full-length/cleaved GSDMD, full-length/cleaved CASP1, full-length/cleaved CASP3, and p-MLKL in HK-2 cells treated with TNF-α supplemented with or without Fludarabine (10 μM). **K-L**) Western blot analysis of the protein levels of (**K**) STAT1, p-STAT1 (Tyr701), p-STAT1 (Ser727), AIM2, and Fibronectin; (**L**) full-length/cleaved GSDMD, full-length/cleaved CASP1, full-length/cleaved CASP3, and p-MLKL from the control groups (NC) and STAT1 knockdown groups (Ri-STAT1) treated with TNF-α. **M**) Representative IF images of STAT1 and p-STAT1 expression in HK-2 cells from the control and TNF-α-treated groups. **N**) Western blot analysis of the protein levels of p-STAT1 (Tyr701) and p-STAT1 (Ser727) in cytoplasmic and nuclear extracts of HK-2 cells treated with TNF-α. **O**) Western blot analysis of the protein levels of STAT1, p-STAT1 (Tyr701), p-STAT1 (Ser727) from the control groups (WT) and STAT1 phospho-site mutant (STAT1-Y701F, and STAT1-S727A) groups treated with TNF-α. **P**) Western blot analysis of the protein levels of STAT1, p-STAT1 (Tyr701), p-STAT1 (Ser727), and AIM2 from the control groups (WT) and STAT1 phospho-site mutant (STAT1-Y701F, and/or STAT1-S727A) treated with TNF-α.

An integrated analysis of multiple transcriptomic datasets (GSE9493, GSE76882, Syn-Allo, and Control-TNF) was conducted to elucidate the mechanisms of AIM2 upregulation, identifying 45 common DEGs (**Figure 7A**). Screening of five predicted AIM2 transcription factors from four databases (**Figure 7B, Table S4**) identified STAT1 for further validation (**Figure 7C**). Western blot analysis revealed significantly elevated p-STAT1 and reduced total STAT1 in CAD renal tissues compared to controls (**Figure 7D**), with a similar pattern observed between the Allo and Syn groups (**Figure 7E**; statistics in **Figure S7A-B**). IHC confirmed the nuclear enrichment of p-STAT1 in the damaged RTECs of both CAD patients and Allo mice (**Figure 7F**). TNF-α stimulation induced site-specific phosphorylation of STAT1: phosphorylation at Y701 preceded phosphorylation at S727, while total STAT1 levels remained unchanged (**Figure 7G-H**). Pharmacological inhibition of phosphorylation using fludarabine significantly reversed the TNF-α-induced upregulation of p-STAT1(Y701), p-STAT1(S727), AIM2, and the fibrotic marker fibronectin (**Figure 7I**), while attenuating the activation of PANoptosis markers (cleaved GSDMD, CASP1, CASP3, and p-MLKL) and the fibrotic indicator fibronectin (**Figure 7J**; statistics in **Figure S7C-D**). STAT1 knockdown further abolished TNF-α-triggered phosphorylation cascades and downstream effector expression (**Figure 7K-L**; statistics in **Figure S7E-F**). Immunofluorescence demonstrated the nuclear translocation of phosphorylated STAT1 upon TNF-α stimulation (**Figure 7M**). Nuclear/cytoplasmic fractionation confirmed that p-STAT1 specifically accumulated in the nucleus (**Figure 7N**; statistics in **Figure S7G-H**). Site-directed mutagenesis of STAT1-Y701F (tyrosine→phenylalanine) abolished Y701 phosphorylation, while STAT1-S727A (serine→alanine) attenuated S727 phosphorylation (**Figure 7O**; statistics in **Figure S7I**). After TNF-α exposure, the Y701F mutation completely abrogated AIM2 upregulation, whereas the S727A mutation partially suppressed this effect (**Figure 7P**; statistics in **Figure S7J**). These findings were further corroborated by Luciferase reporter assays (**Figure S8A**). These results establish that TNF-α activates STAT1-mediated AIM2 transcription through Y701-dominated dual-site phosphorylation, thereby driving tubular epithelial PANoptosis.

### TNF-α induces STAT1 phosphorylation at Tyr701 to directly binds to the promoter region to promote AIM2 transcription

**Figure 8:**
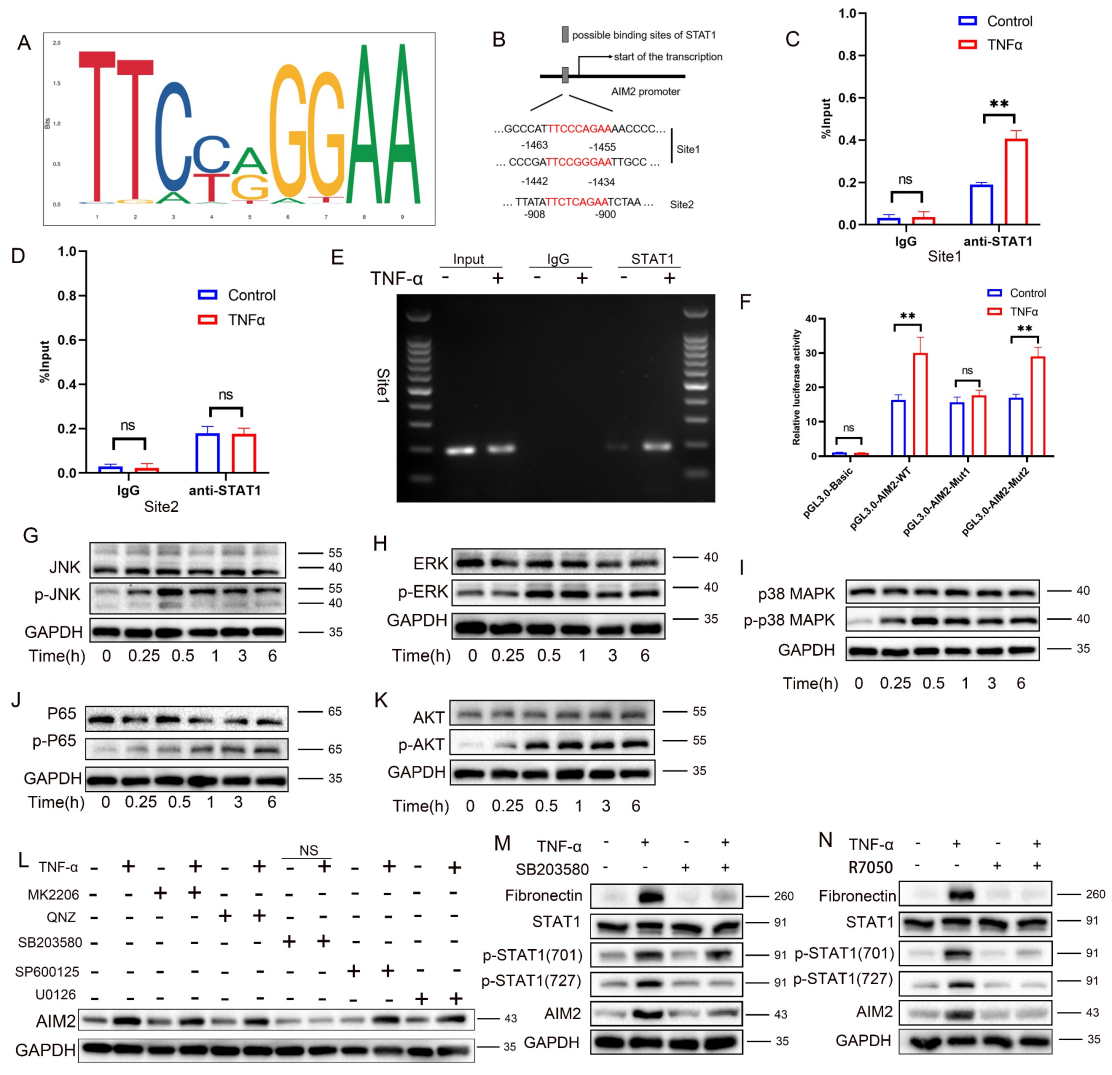
** TNF-α induces STAT1 phosphorylation at Tyr701 to directly bind the AIM2 promoter and enhance transcription, and phosphorylation at Ser727 via the p38/MAPK pathway further augments AIM2 transcription. A**) STAT1 DNA-binding motif obtained from JASPAR. **B**) Predicted STAT1-binding sites within the AIM2 promoter (JASPAR). **C-E**) HK-2 cells were treated with TNF-α (100 ng/mL) or vehicle for 24 h, then subjected to ChIP using anti-STAT1 antibody; IgG served as the negative control. (**C**, **D**) qPCR quantification of STAT1 enrichment at site 1 and site 2. (**E**) Agarose-gel verification of site-1 amplification. **F**) Relative luciferase activity of cells transfected with pGL3-AIM2-WT reporter plasmid and mutant plasmids (pGL3-AIM2-MUT1 or pGL3-AIM2-MUT2) after treatment ± TNF-α. **G-K**) Western blot analysis of the protein levels of JNK, p-JNK, ERK, p-ERK, p38MAPK, p-p38MAPK, p65, p-p65, AKT, and p-AKT in HK-2 cells treated with TNF-α (100 ng/mL) for 0, 0.25, 0.5, 1, 3, and 6 h. **L**) Western blot analysis of AIM2 in HK-2 cells treated with TNF-α supplemented with or without (MK-2206 [AKT inhibitor], QNZ [NF-κB inhibitor], SB203580 [p38MAPK inhibitor], SP600125 [JNK inhibitor], or U0126 [MEK/ERK inhibitor]) for 24 h. **M**) Western blot and quantitative analysis of Fibronectin, STAT1, p-STAT1 (Tyr701), p-STAT1 (Ser727), and AIM2 in HK-2 cells treated with TNF-α supplemented with or without SB203580. **N**) Western blot analysis of Fibronectin, STAT1, p-STAT1 (Tyr701), p-STAT1 (Ser727), and AIM2 in HK-2 cells treated with TNF-α supplemented with or without R7050 (TNF-α Antagonist).

To further elucidate the mechanism by which STAT1 regulates AIM2 expression, the STAT1 DNA-binding motif from the JASPAR database was analyzed (**Figure 8A**). Prediction *via* JASPAR identified STAT1-binding sites within the AIM2 promoter region, with the top three highest-scoring sites selected for study (**Figure 8B and Table S5**). Among these, the -1463 to -1455 and -1442 to -1434 sites, adjacent to each other, were designated as Site 1 (the highest-scoring site), while the site at -908 to -900 was defined as Site 2. Chromatin immunoprecipitation PCR (ChIP-PCR) demonstrated that STAT1 directly bound to Site 1 of the AIM2 promoter upon TNF-α stimulation (**Figure 8C-D**), with agarose-gel verification of site-1 amplification (**Figure 8E**). Luciferase reporter assays further revealed that mutation of Site 1 abolished STAT1-mediated transcriptional activation compared to the wild-type construct, while mutation at Site 2 had no significant impact (**Figure 8F**). These results collectively indicate that TNF-α-induced phosphorylation of STAT1 at Y701 enables its direct binding to the AIM2 promoter, driving transcriptional upregulation.

### TNF-α induces STAT1 phosphorylation at Ser727 via the p38/MAPK pathway further augments AIM2 transcription

Western blot analysis revealed concomitant activation of the MAPK pathway (p-JNK, p-ERK, and p-p38), the NF-κB pathway (p-p65), and the AKT/mTOR pathway (p-AKT) following TNF-α stimulation (**Figure 8G-K**; statistics in **Figure S8B-F**), thereby delineating the activation of inflammatory pathways induced by TNF-α. Pharmacological inhibition experiments showed that only the p38 MAPK inhibitor (SB203580) reversed TNF-α-mediated AIM2 upregulation (**Figure 8L**; statistics in **Figure S8G**). Further analysis revealed that SB203580 suppressed TNF-α-induced increases in AIM2, p-STAT1(Ser727), and the fibrosis marker fibronectin, but did not attenuate p-STAT1(Tyr701) (**Figure 8M**; statistics in **Figure S8H**). Conversely, the TNF-α receptor inhibitor (R7050) abolished the TNF-α-triggered upregulation of AIM2, p-STAT1(Tyr701), p-STAT1(Ser727), and fibronectin (**Figure 8N**; statistics in **Figure S8I**). These results collectively indicate that TNF-α enhances AIM2 transcription through phosphorylation of STAT1 at Ser727 *via* the p38 MAPK pathway. Additional animal experiments confirmed that R7050 significantly attenuated PANoptosis in renal allografts and mitigated the progression of renal interstitial fibrosis (**Figure S9A-E**).

### PANoptotic RTECs promote EMT through paracrine secretion of IL-6 and TGF-β1

**Figure 9:**
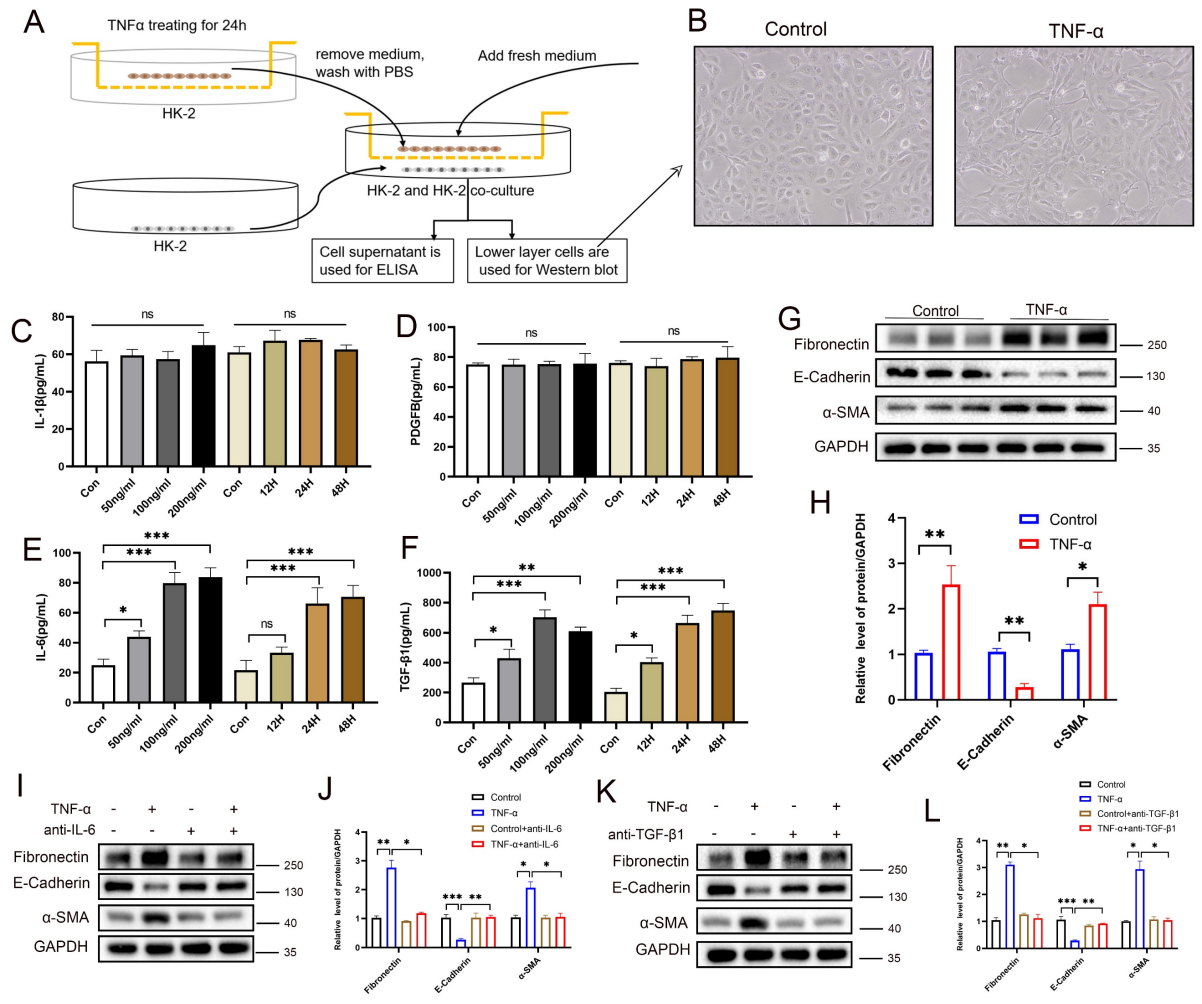
** PANoptotic RTECs promote EMT through paracrine secretion of IL-6 and TGF-β1. A**) HK-2 and HK-2 co-culture system diagram. **B**) Representative light-microscopy images of the lower-layer HK-2 cells in the co-culture system. **C-F**) Levels of IL-1β, PDGF-BB, IL-6, and TGF-β1 in co-culture supernatants measured by ELISA. **G-H**) Western blot and quantitative analysis of the protein levels of fibronectin, E-cadherin, and α-SMA in lower-layer HK-2 cells.** I-J**) Western blot and quantitative analysis of the protein levels of fibronectin, E-cadherin, and α-SMA in the lower-layer HK-2 cell line in the co-culture system treated with TNF-α supplemented with or without anti-IL-6. **K-L**) Western blot and quantitative analysis of the protein levels of fibronectin, E-cadherin, and α-SMA in the lower-layer HK-2 cell line in the co-culture system treated with TNF-α supplemented with or without anti-TGF-β1. *P < 0.05, **P < 0.01, ***P < 0.001, ns indicates no significance.

To investigate the paracrine effects of tubular epithelial PANoptosis on neighboring cells, an HK-2-HK-2 co-culture system was established (**Figure 9A**). Morphological analysis revealed a subset of cells transitioning from a cobblestone-like to a spindle-shaped morphology, indicating EMT and cell fibrosis (**Figure 9B**). ELISA results showed no significant changes in IL-1β or PDGFβ levels, but a significant elevation of IL-6 and TGF-β1 in the co-culture supernatants (**Figure 9C-F**). Western blot analysis confirmed the downregulation of the epithelial marker E-cadherin, alongside the upregulation of the fibrotic markers α-SMA and fibronectin in adjacent cells (**Figure 9G-H**), suggesting activation of EMT. Neutralizing antibody interventions demonstrated that anti-IL-6 significantly rescued E-cadherin suppression and α-SMA/fibronectin elevation (**Figure 9I-J**), while anti-TGF-β1 elicited equivalent rescue effects (**Figure 9K-L**). These results demonstrate that PANoptotic RTECs promote EMT in neighboring cells *via* the secretion of IL-6 and TGF-β1. Moreover, co-culture experiments revealed that TNF-α-stimulated HK-2 cells promote the fibrotic activation of NRK-49F (**Figure S10A-G**) and THP-1 (**Figure S10H-N**) cells *via* IL-6 and TGF-β secretion.

## Discussion

**Figure 10:**
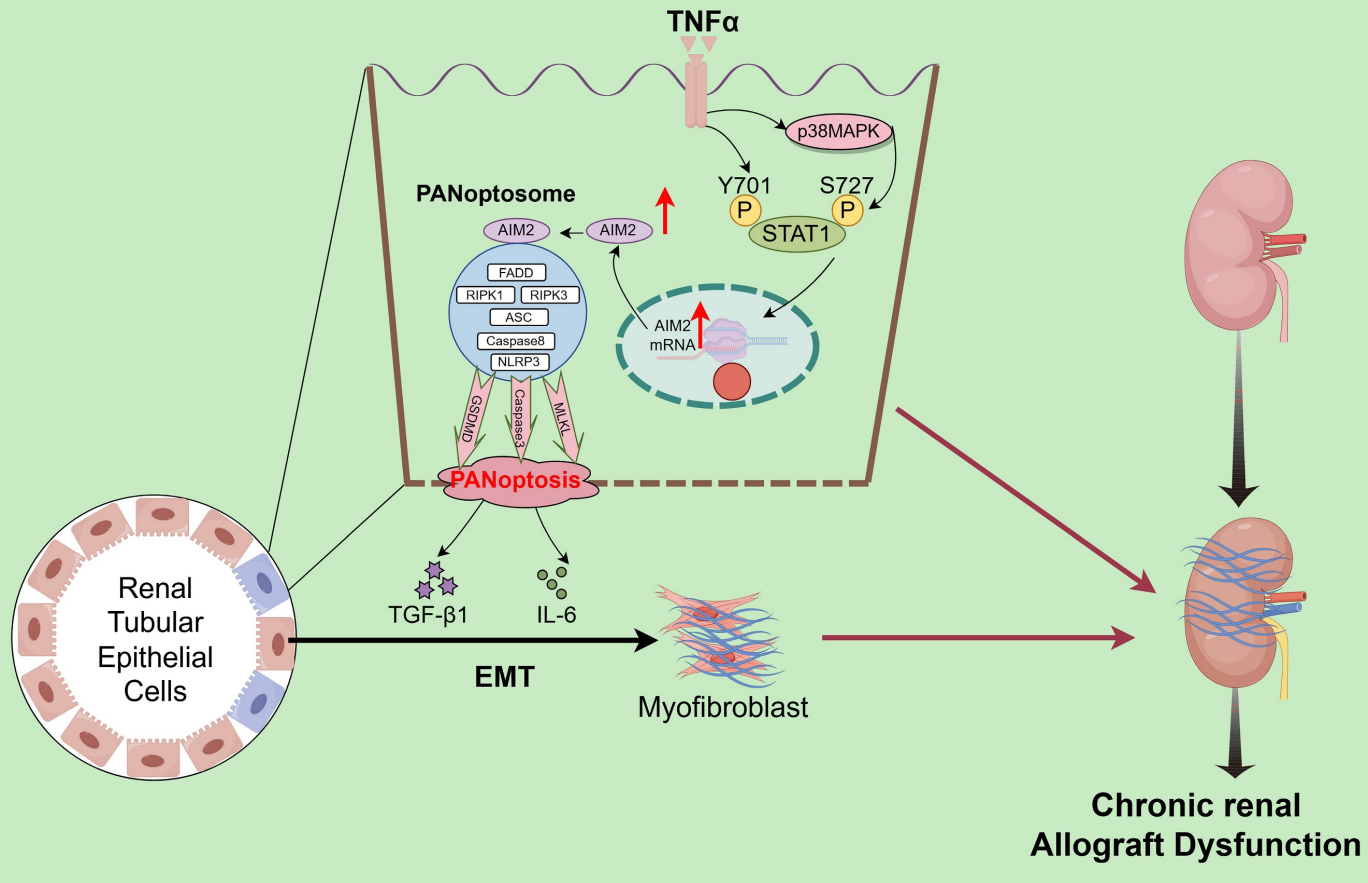
** Schematic description of the molecular mechanism.** TNF-α drives tubular epithelial injury *via* the direct induction of STAT1 Y701 phosphorylation and p38 MAPK-mediated STAT1 phosphorylation at S727, cooperatively upregulating AIM2 to execute PANoptosis. PANoptotic cells secrete IL-6 and TGF-β, inducing EMT in adjacent tubular cells, thereby collectively promoting renal allograft interstitial fibrosis.

​ Interstitial fibrosis in renal allografts is a major cause of graft loss post-transplantation. Effective antifibrotic strategies are essential to delay allograft dysfunction and improve patient quality of life but remain significant clinical challenges. scRNA-seq quantified the abundance of various PCD modalities in CAD tissues, identifying PANoptosis as the central driver of tubular epithelial injury. Validation across clinical specimens and animal models confirmed PANoptosis activation and highlighted the critical regulatory role of the AIM2 inflammasome. Mechanistically, TNF-α in the CAD microenvironment induces tubular epithelial injury by directly triggering STAT1 Y701 phosphorylation and p38 MAPK-mediated STAT1 phosphorylation at S727, which cooperatively upregulates AIM2 to execute PANoptosis. Notably, PANoptotic cells secrete IL-6 and TGF-β, which promote EMT in adjacent tubular cells (**Figure 10**). The TNF-α/STAT1/AIM2 axis-mediated PANoptosis and its paracrine signaling contribute to renal allograft interstitial fibrosis. Our study systematically elucidates the role of PANoptosis in transplant tubular injury, establishes its central role in CAD-associated fibrogenesis, and identifies novel therapeutic targets.

Persistent chronic inflammation after renal transplantation is a core pathological driver of allograft interstitial fibrosis, with inflammatory cytokine cascades playing pivotal roles 27, 28. Activated inflammatory cells release multiple cytokines that mediate fibrogenesis through interconnected pathways. TNF-α 29, 30 and IL-1β 31 directly damage RTECs, induce adhesion molecule expression (e.g., ICAM-1), and recruit neutrophils, monocytes, and macrophages, thereby establishing a self-amplifying injury-inflammation positive feedback loop 32, 33. IL-6 activates the JAK/STAT signaling pathway to promote interstitial fibroblast proliferation 34, while TGF-β, acting as a central link between inflammation and fibrosis, is induced by upstream factors such as TNF-α and IL-1β. TGF-β drives fibroblast-to-myofibroblast differentiation *via* Smad-dependent signaling 35-37. Myofibroblasts then become the primary effectors of pathological ECM deposition, overproducing type I/III collagens. This vicious inflammation-fibrosis cycle, initiated by chronic inflammation, amplified through cytokine cascades, and culminating in aberrant ECM accumulation, progressively disrupts renal architecture and function 38, 39. While our previous work demonstrated TNF-α's role in promoting allograft fibrosis 24, 26, the current study provides a deeper understanding of its molecular mechanisms in tubular epithelial injury.

In the progression of chronic interstitial fibrosis in renal allografts, RTECs function not only as targets of injury but also as active contributors, with multiple PCD modalities playing pivotal roles. PANoptosis, a pathway integrating pyroptosis, apoptosis, and necroptosis, is activated in chronic inflammatory environments 40. Specifically, NLRP3 inflammasome activation cleaves caspase-1, facilitating IL-1β/IL-18 maturation and GSDMD-mediated plasma membrane pore formation, leading to osmotic cell rupture (pyroptosis) 41; caspase-3/caspase-8 cascades mediate apoptosis; and the RIPK1/RIPK3/MLKL axis drives necroptosis 42. The massive release of damage-associated molecular patterns (DAMPs) and inflammatory factors recruits neutrophils and macrophages, perpetuating a chronic inflammatory microenvironment that persistently activates interstitial fibroblasts, ultimately leading to pathological ECM deposition 43. Our study demonstrates the concurrent activation and crosstalk of these PCD pathways in RTECs, whose collective injurious effects cannot be solely attributed to any single pathway, jointly promoting renal allograft interstitial fibrosis.

AIM2, a crucial cytosolic DNA sensor, acts as the master initiator of PANoptosome assembly. Upon recognizing aberrant double-stranded DNA (dsDNA), such as pathogen-derived DNA or nuclear DNA released during cellular damage, AIM2 specifically binds to dsDNA through its C-terminal HIN domain 44. This binding induces conformational changes that expose the N-terminal PYRIN domain, recruiting the adaptor protein ASC to form the AIM2 inflammasome, which subsequently assembles the functional PANoptosome 10. The PANoptosome executes PCD through dual mechanisms: Activated caspase-1 cleaves pro-IL-1β and pro-IL-18, initiating pyroptotic inflammation, while also activating caspase-3-dependent apoptosis and RIPK3-mediated necroptosis 45. Our study demonstrates that TNF-α transcriptionally upregulates AIM2 expression in RTECs. Elevated AIM2 further activates downstream effectors such as CASP1, CASP3, and MLKL, driving the coordinated activation of pyroptosis, apoptosis, and necroptosis.

The phosphorylation-mediated transcriptional regulation of AIM2 by STAT1, a member of the STAT family, represents a key molecular event linking inflammatory signaling to immune responses 46, 47. STAT1 phosphorylation is governed by upstream cytokine signals: Conventional studies have shown that IFN-γ induces conformational changes in cell surface receptors, recruiting JAK kinases (JAK1/JAK2) to phosphorylate STAT1 at Y701 48, 49, which then enables its nuclear translocation for transcriptional activity. Some studies also indicate that phosphorylation of STAT1 at S727 plays an important role in its transcriptional regulation 50. Our study reveals that the inflammatory cytokine TNF-α activates dual-site phosphorylation of STAT1, directly inducing Y701 phosphorylation while activating S727 phosphorylation (p-STAT1[S727]) through the p38 MAPK pathway. Phosphorylated STAT1 synergistically translocates to the nucleus, binds to specific *cis*-regulatory elements in the AIM2 promoter, and upregulates AIM2 expression. This drives tubular epithelial PANoptosis, with Y701 phosphorylation being essential for transcriptional initiation and S727 phosphorylation enhancing AIM2 upregulation. PANoptotic cells secrete IL-6 and TGF-β, which induce EMT in adjacent cells, thereby collectively promoting renal allograft interstitial fibrosis.

​​ The limitations of our study include its focus on PANoptosis mechanisms in tubular epithelia, with downstream pathways linking PANoptosis to EMT/fibrosis requiring further exploration. Additionally, *in vivo* validation of AIM2 knockout remains incomplete. Future studies should target critical nodes, such as STAT1 and AIM2, to develop therapeutic strategies for combating allograft fibrosis.

## Conclusions

Within the chronic inflammatory microenvironment of CAD, TNF-α drives tubular epithelial injury through dual mechanisms: direct induction of phosphorylation at STAT1 Y701 and activation of the p38 MAPK pathway to mediate phosphorylation at STAT1 S727. Cooperative phosphorylation at these sites enhances STAT1 transcriptional activity, upregulates AIM2 expression, and triggers PANoptosis. PANoptotic cells secrete IL-6 and TGF-β, which induce EMT in adjacent cells, thereby collectively promoting renal allograft interstitial fibrosis.

## Supplementary Material

Supplementary methods, figures and tables.

## Abbreviations

CAD: chronic renal allograft dysfunction
TNF-α: tumor necrosis factor-alpha
STAT1: signal transducer and activator of transcription 1
AIM2: absent in melanoma 2
EMT: epithelial-mesenchymal transition
RTECs: renal tubular epithelial cells
PCD: Programmed cell death
IL-6: interleukin-6
TGF-β1: transforming growth factor-β1
ChIP: Chromatin immunoprecipitation
IHC: Immunohistochemistry
IF: Immunofluorescence
ELISA: Enzyme-Linked Immunosorbnent Assay
GO: Gene Ontology
KEGG: Kyoto Encyclopedia of Genes and Genomes
PAS: Periodic Acid-Schiff
NLRP3: NOD-like receptor thermal protein domain associated protein 3
GSDMD: gasdermin D
CASP1: Caspase1
CASP3: Caspase3
CASP8: Caspase8
RIPK1: Receptor Interacting Protein Kinase-1
RIPK3: Receptor Interacting Protein Kinase-3
t-MLKL: total-mixed lineage kinase domain like pseudokinase
p-MLKL: phosphorylated MLKL
