# Supplementary material for: Dual Phosphorylation of STAT1 at Y701/S727 by TNFα Drives AIM2-Mediated PANoptosis of Renal Tubular Epithelial Cells and Fibrotic Progression in Renal Allografts
Source: Int J Biol Sci. 2026 Jan 1;22(2):582–600. doi: 10.7150/ijbs.123441 (PMC12780947; doi:10.7150/ijbs.123441)
Supplement: Supplementary file 1 — Supplementary methods, figures and tables. [file ijbsv22p0582s1.pdf]

Supplementary material 1: The detailed operational procedures of animal models

The left kidney of donor mice was transplanted into the peritoneal cavity of recipient mice, with simultaneous intraoperative nephrectomy of the recipient's native right kidney. On day 7 post-transplantation, the contralateral (left) kidney of recipient mice was additionally excised. During surgery, mice were anesthetized with inhaled isoflurane, and the total ischemic time averaged 40–60 minutes. To prevent acute rejection, recipient mice received oral tacrolimus at a dosage of 1 mg/kg once daily for 7 days, followed by 1 mg/kg once weekly for the subsequent 4 weeks. For TNF receptor (TNFR) inhibition, Syn group and Allo group animals received intraperitoneal injections of R7050 (10 mg/kg; HY-110203) once daily for 7 days, followed by once weekly for the subsequent 4 weeks. At 16 weeks post-transplantation, mice were euthanized, and transplanted kidney tissues were either fixed in paraffin or stored in liquid nitrogen.

Table S1: Primer sequences

AIM2-Forward	TGGCAAAACGTCTTCAGGAGG
AIM2-Reverse	AGCTTGACTTAGTGGCTTTGG
ZBP1 -Forward	AACATGCAGCTACAATTCCAGA
ZBP1-Reverse	AGTCTCGGTTCACATCTTTTGC
Ripk1-Forward	GGGAAGGTGTCTCTGTGTTTC
Ripk1-Reverse	CCTCGTTGTGCTCAATGCAG
NLRP12-Forward	GGGGCTTGTCAGGAGATGG
NLRP12-Reverse	AGTCCCTGGCATAGTAACCTC

Table S2: The details of primary antibodies for Western blot assay, IHC and IF

The primary antibodies for Western blot assay

Fibronectin (Proteintech, 15613-1-AP,1:2000); E-cadherin (SAB,40860,1:1000); α-SMA (SAB,40482,1:400); NLRP3(CST, 15101,1:1000); GSDMD(CST, 39754,1:1000); Cleaved GSDMD(CST, 36425,1:1000);Cleaved CASP1 (CST, 89332, 1:1000); Caspase-1 (CST, 98033, 1:1000); cleaved CASP3 (Affinity,AF7022,1:1000) , CASP8 (Proteintech, 66093-1-Ig, 1:2000); RIPK1 (Proteintech, 17519-1-AP, 1:100); RIPK3 (Proteintech, 17563-1-AP, 1:1000); MLKL (Proteintech, 66675-1-Ig, 1:5000) ;phosphorylated MLKL (p-MLKL) (Affinity,AF7420,1:1000) ;AIM2 (Proteintech, 20590-1-AP, 1:1000); TNFα (Proteintech, 17590-1-AP,1:1000);STAT1(Proteintech, 10144-2-AP, 1:2000); p-STAT1 (Tyr701) (CST, 9167,1:1000);p-STAT1 (Ser727) (CST, 8826,1:1000); JNK (CST, 67096,1:1000); p-JNK (CST, 9251,1:1000); ERK (CST, 4695,1:1000); p-ERK (CST, 4370,1:2000); p38MAPK (CST, 9212,1:1000); p- p38MAPK (CST, 4511,1:1000); p65 (CST, 4764,1:1000); p-p65 (CST, 3033,1:1000); AKT(CST, 9272,1:1000) ;p- AKT(CST, 4060,1:2000); GAPDH (Proteintech, 60004-1-Ig, 1:10000).

The primary antibodies for IHC

Primary antibodies for IHC were as follows: Cleaved GSDMD(CST, 36425,1:200);GSDMD(CST, 69469,1:50);Cleaved CASP1 Affinity,AF4005,1:100); CASP1 (CST, 98033, 1:200); CASP3 (CST, 9662,1:100); cleaved CASP3 (Affinity,AF7022,1:100) , CASP8 (Proteintech, 66093-1-Ig, 1:100); RIPK1 (Proteintech, 17519-1-AP, 1:1000); RIPK3 (Proteintech, 17563-1-AP, 1:100); p-MLKL(Abcam, ab187091,1:200) ;AIM2 (Proteintech, 20590-1-AP, 1:50); TNFα (Proteintech, 17590-1-AP,1:100); p-STAT1 (Tyr701) (CST, 9167,1:400).

The primary antibodies for IF

Cleaved GSDMD(CST,36425,1:200); Cleaved CASP1 (Affinity, AF4005,1:100); cleaved CASP3(CST, 9661,1:200); p-MLKL(Abcam, ab187091,1:100); AQP1 (Proteintech, 20333-1-AP, 1:100); STAT1(Proteintech, 10144-2-AP, 1:200); p-STAT1 (Tyr701) (CST, 9167,1:400).

Table S3: AIM2 promoter sequence and primer sequence designed based on site 1 and site 2

>hg38_knownGene_ENST00000368130.9 range=chr1:159076767-159078766 5'pad=0 3'pad=0 strand=- repeatMasking=none

CTCCGTGAGACTGTTAACAAACCTCCTTTTTCAGCACAGTTGCCTCCTTTCCCTTCTGTGTCTTGTGTCACCAAGTTG
GGTATCTCTCTGATTAAATTTCTTGGTAAAACAAAGCTGGACGATGAGTACTTGTCTCTCTGCCCCAGGTGCAGCAG
TGTACCTGCTCCTCATTGAGACCACTTCCCAGCTTAATCACTTAACTCACCTTTTACAGGACCTGAAGCCTCAAATCA
GTACGCCTCTCTGGGGTTGGTGCTTGTTGGGTTCTTCCTCAACAGGGAGCTTTCTCTCGTTTACTCAGTAGGTTACCA
AATTCATACACTTCACATCTTTTTAATTCTTACATATTGTTCTCTTTGGTGTTCTTTCGCCTTGTGTGATCCACTCATTCA
TGCATTCACTCATTCAACGAATATTTTCTGAACACCTTCTCTGTGCCTGGCACTGTGTCAGGTGAAGTGGATGCAGT
GAGCCAGTGGGCAGAAATCTCTTCCCTCATGTGCCTTACATTTTAGGAGCATGAGGCAGAGAATAAACAAACAAAT
GACATATACCGTATGTCAGATGATGGTCAGTAACACGTGTGGGAAAAGATAATGTGGGGAAGAGGTAGAGAACATG
CTGGTCAGAGAAGCCCCACAGATAAGGTGCTATTTGAGCTGAAAGCTGGCAAAAGTGTAGAAACAAGCCTTGCAGT
AGAAAGTGAAGGAGGTCCTGCAGCTCTGGGCTGATTACCTGGGCATGGTTAGGTGCTCTTCCTCTGCTCCTTGAGCA
TCTTGAGCATGCTTTAATTATAACACTTATCATCATGTCCTATTGTGATTATTAAATCACATGTCTGTTTTCATGACTTG
GCAGAGCCGCTCAACATCTGGAACTTTGTCTTACTAACTTTTATATTCTCAGAATCTAATACATCATATTAGATGTTCA
GTGCATAATTGTTAATTCAATGAATAAGAAAGGAAAACCTCAAGTAAATTGCTGACTAGAATCAAGCCCTCAAGCTC
TGAATTTAGGGTTAGGAGCCTGGGTTAGGAAGAAAGACTGTATATATGCTTTTGAGGCAAGCAGTCAGTGAGGGAAT
GAGGCATGATTACTTGCAGGTGGCTACAAGCAATAAAAATGCGGTCAAAAAGGTTAGCAGAGGGATTGCCCCACTA
GTAGATAGGAACTGTTACAACAGCCTGTGACAATGAAGACAGATTGCAAACTTCCTACTTAGGCACAGATGAGAAT
GTAACACAGAAGAAACTGGCCGCAACTGGTTAAAGCCAAGATGGTCGAAAACTTGGCTGACTGCTGACCCTTAGCT
TCATTATGCCCTTAATATCATAAAACTTCCATGGGGAAATTCCCACTCCCATCATGCACCTGACGCCATGACAGTTCC
GGATTAACCATCTTTAGTCAAGAAAAGGGTGGCACCCCGATTCCGGGAATTGCCCGCCCATTTCCCAGAAAACCCCT
CCCCTTGATTACAGAGTACTCCATACCTTCATTGTGCTTATTCATATAGTACGTGAACCACATTGCCTGTGACTCAAAT
CTTTGAGTGCCTGCACTCCTCCCTTGAGGGTGTCGTTGGTTTTGCTCCACAATAAAATGTCTATACTTTCGCTTGGGT
CTCATTTTCAGATTCTCTTATGCTTTGAAGACAAGAACCTGCACTGCCCTACTGGCAACACTTTTAGATACCCTCTGA
GATCAGTGTGCTTGTTTATACCAGAAAAATGTCATCACCAGGCCCCTTAGTCACAAAGTGTATCAAGAAGTCATCTG
CGGTCATTTTTTACAGTTTCTAGGCCACTTTCTGTTTCCAATCAGTGTAGTTCCTATGTGGCATGACTCAGATACAAGT
TTCTATAGACATTTTCTTCTGTGGCTGCTAGTGAGAACCCAAACCAGCTCAGCCAATTAGAGCTCCAGTTGTCACTCC
TACCCACACTGGGCCTGGGGGTGAAGGGAAGTGTTTATTAGGGGTACATGTGAAGCCGTCC

site 1

Forward：CGGATTAACCATCTTTAGTCAAGA（GC：37.5%；Tm：57.9℃）

Reverse：GAAGGTATGGAGTACTCTGTAATCAAG（GC：40.7%；Tm：57.8℃）

site 2

Forward：AGCCGCTCAACATCTGGAAC（GC：55%；Tm：60℃）

Reverse：AGGGCTTGATTCTAGTCAGCAAT（GC：43.5%；Tm：59.4℃）

Table S4: AIM2 transcription factors from hTFtarget, KnockTF, JASPAR and GTRD

**hTFtarget**									
CTCF	ATF2	BACH2	BATF	BCL11A	BCL3	BCL6	BRD4	CDK9	CEBPA
CEBPB	CREB1	EBF1	EP300	FLI1	FOXM1	FOXP1	GATA2	GATA3	HDAC2
IRF1	IRF4	JUND	MAFK	MAX	MAZ	MED1	MEF2A	MTA3	MYB
NFIC	NFYB	PAX5	PBX3	POLR2A	RAD21	RELA	RUNX1	RUNX3	SIX5
SP1	SPI1	SPIB	STAG1	STAT1	STAT3	STAT4	STAT5A	STAT5B	TAL1
TBX21	TCF12	TCF3	TFAP4	VDR	YY1	ZNF143	ETS1	RBPJ	ESR1
ESR2	ATF3	CBX3	ELF1	ELK1	FOS	FOSL1	GATA1	GTF3C2	HCFC1
JUN	JUNB	MAFF	MEIS1	NR2F2	POLR3A	STAT2	TBL1XR1	TBP	TEAD4
TRIM28	CREBBP	FOSL2	REST	RXRA	SMC3	TAF1	USF1	FOXA1	KDM5B
MYC	TFAP2C	CENPA	SMC1A	GATA4	NANOG	OTX2	POU5F1	SRF	KLF4
RYBP	ZNF263	LMNB1	SMAD3	CIITA	DUX4	HDAC6	MYH11	SMAD1	AR
MRE11A	GATA6	BCOR							
**KnockTF**									
POU5F1	PROX1	NR2F2	TP53	ZIC2	FOXM1	POLR3A	HSF1	STAT3	TCF4
TP63	HIC1	STAT1	MYC	MITF	TWIST1	MSX1	ZIC5	PTEN	ETV5
TFAP2C	SMAD3	SOX17	MYB	POLR2G	AGO2	PTBP1	SAFB2	HNRNPUL1	HNRNPL
U2AF2	ZNF395	NR2C2	HMGN1	AR	ARID1A	BCL6	FLI1	FOXO1	GATA4
GFP	HMGA1	MEF2D	MTA2	NMI	RELB	SOX2	STAT6	ZNF746	CTCF
NUP35									
**JASPAR**									
ZNF135	PRDM9	ZNF75D	SPIB	ZNF75A	ZNF257	ZNF701	ZNF530	ZNF281	ZNF331
ZFP14	ZNF460	ZNF454	ZNF184	ASCL1	ZNF148	NR2C2	ZNF684	MYOD1	ETV5::FOXI1
SP1	TFDP1	E2F6	ZNF449	PATZ1	NFIB	SP4	ZKSCAN5	SRF	IRF2
NHLH1	GABPA	ESR2	TFAP4	ZNF816	NR1I2	ZNF740	SIX1	ELF3	EHF
ZNF766	STAT3	ERF::FOXI1	MAZ	ZNF770	NFKB2	FOXJ2::ELF1	CTCF	PHOX2B	PPARG
MYOG	ZBED4	ZNF93	NR5A1	ZNF667	STAT1::STAT2	MAFF	SPIC	SP2	NKX2-8
TCF4	KLF9	ELF1	ETV2::FOXI1	TFAP2A	STAT1	ZNF175	KLF4	ZBTB7B	KLF16
CTCFL	KLF15	MYF5	NR1I3	KLF17	ZNF784	NKX2-4	TBX21	NFYB	TFAP2C
ZNF263	TCF12	SNAI1	TCF3	ZNF677	ETV1	ETV2::HOXB13	KLF10	ZBTB24	SNAI3
HES1	KLF14	FOXO1::ELK1	NKX2-3	NFYA	ZNF574	ZNF324	SP8	FLI1::FOXI1	ZBTB26
DPRX	FLI1::DRGX	ONECUT1	E2F8	SP9	TBX3	OSR2	MSANTD3	JUND	MAFK
ZNF416	ERF::FOXO1	KLF12	PKNOX1	FOXO1::ELF1	JUN::JUNB	TCFL5	ZBTB7C	OSR1	PRDM1

NFYC	EBF1	PLAGL2	EGR4	KLF1	RARA::RXRG	MSC	PPARA::RXRA	KLF2	ZNF417
ZNF768	ETV2	ETV5::DRGX	ATOH7	SNAI2	FOS::JUND	PAX6	ZKSCAN1	EGR1	PBX3
FOSL2::JUN	FOSL1::JUN	PBX1	FOSL2::JUNB	EBF3	VSX2	VSX1	VAX1	SRY	PRRX2
NOTO	MEOX1	LHX9	LBX1	HMBOX1	ESX1	FOXD3	ETV7	FOXP2	MYC
**GTRD**									
AFF1	AHR	AR	ARNT	ASCL1	ATF2	ATF3	ATF7	BACH2	BATF
BATF3	BCL11A	BCL6	BMI1	BRD2	BRD3	BRD4	BRD9	CBFA2T3	CBX2
CBX3	CCAR2	CCNT2	CDK8	CDK9	CDKN1B	CEBPA	CEBPB	CEBPD	CHD1
CHD8	CIITA	CREB1	CREB3L1	CREBBP	CREM	CTCF	DAXX	DDX20	DPF2
DUX4	E2F1	E2F6	E2F8	EBF1	EBP	EGR1	ELF1	ELK1	ELK4
EMSY	EP300	ERG	ESR1	ESR2	ETS1	ETV5	ETV6	EZH2	FLI1
FOS	FOSL1	FOSL2	FOXA1	FOXA2	FOXK2	FOXO1	FOXP1	GABPA	GATA1
GATA2	GATA3	GATA4	GATAD2B	GFI1	GFI1B	GTF3C2	HBZ	HCFC1	HDAC1
HDAC2	HEY1	HIC1	HIF1A	HNF4A	IKZF1	INO80	INTS11	INTS13	IRF1
IRF2	IRF4	JUN	JUNB	JUND	KDM1A	KDM5B	KDM6A	KLF1	KLF4
KLF5	KMT2A	KMT2B	LDB1	LMNA	LMNB1	LYL1	MAF	MAFK	MAX
MBD4	MCM7	MECP2	MED12	MEF2B	MEIS1	MEIS2	MEIS3P1	MITF	MYB
MYC	MYCN	NCOA3	NCOR1	NEUROD1	NFE2	NFIC	NFKB1	NFKB2	NFKBIA
NFRKB	NFYA	NKX2-1	NR2F1	NR2F2	NR2F6	NR3C1	NR4A1	ORC2	OTX2
PADI2	PARP1	PAX5	PBX3	PGR	PHF8	POU2F2	RAD21	RARA	RBBP5
RBFOX2	RBM25	RBPJ	RCOR1	REL	RELA	RELB	REST	RING1	RNF2
RUNX1	RUNX1T1	RUNX2	RUNX3	RXRA	SAP30	SETDB1	SIRT1	SIRT6	SIX5
SKI	SMAD1	SMAD3	SMAD5	SMARCA4	SMARCB1	SMARCC2	SMC1A	SMC3	SNAI2
SP1	SPI1	SPIB	SREBF2	SRF	SS18	SSRP1	STAG1	STAT1	STAT2
STAT3	STAT4	STAT5A	STAT5B	STAT6	SUMO2	SUPT5H	SUZ12	TBP	TBX21
TCF12	TCF3	TCF4	TEAD4	TFAP2A	TFAP2C	TFDP1	TLE3	TP53	TP63
TRIM24	TRIM28	TWIST1	USF1	VDR	VEZF1	WT1	XRCC5	YY1	ZBED1
ZBTB33	ZBTB7A	ZFP36	ZMYND8	ZNF143	ZNF148	ZNF24	ZNF264	ZNF366	ZNF395
ZNF597	ZNF766	ZSCAN5A							

Table S5: The binding site of STAT1 in the AIM2 promoter region identified by JASPAR

Matrix ID	Score	Relative score	Sequence ID	Start	End	Strand	Predicted sequence
MA0137.4	15.47562	0.998737	hg38_knownGene_ENST00000368130.9	1434	1442	+	ttccgggaa
MA0137.4	15.06818	0.992784	hg38_knownGene_ENST00000368130.9	1455	1463	-	ttctgggaa
MA0137.4	10.77909	0.930114	hg38_knownGene_ENST00000368130.9	900	908	-	ttctgagaa
MA0137.4	8.698243	0.899709	hg38_knownGene_ENST00000368130.9	1434	1442	-	ttcccggaa
MA0137.4	4.935602	0.844732	hg38_knownGene_ENST00000368130.9	209	217	+	ttacaggac
MA0137.4	4.529453	0.838797	hg38_knownGene_ENST00000368130.9	98	106	+	ttcttggta
MA0137.4	4.502993	0.838411	hg38_knownGene_ENST00000368130.9	1818	1826	-	tgattggaa
MA0137.4	4.409154	0.837039	hg38_knownGene_ENST00000368130.9	1455	1463	+	ttcccagaa
MA0137.4	4.001714	0.831086	hg38_knownGene_ENST00000368130.9	900	908	+	ttctcagaa
MA0137.4	3.681276	0.826404	hg38_knownGene_ENST00000368130.9	405	413	+	ttcaacgaa
MA0137.4	3.681276	0.826404	hg38_knownGene_ENST00000368130.9	949	957	+	ttcaatgaa
MA0137.4	3.505931	0.823842	hg38_knownGene_ENST00000368130.9	1961	1969	+	tgaagggaa
MA0137.4	3.400978	0.822309	hg38_knownGene_ENST00000368130.9	98	106	-	taccaagaa
MA0137.4	2.999157	0.816437	hg38_knownGene_ENST00000368130.9	986	994	-	ttctagtca
MA0137.4	2.911139	0.815151	hg38_knownGene_ENST00000368130.9	1723	1731	-	ttctggtat
MA0137.4	2.828783	0.813948	hg38_knownGene_ENST00000368130.9	436	444	-	tgccaggca
MA0137.4	2.475364	0.808784	hg38_knownGene_ENST00000368130.9	1097	1105	-	tgcaagtaa
MA0137.4	2.352078	0.806982	hg38_knownGene_ENST00000368130.9	181	189	-	agctgggaa
MA0137.4	1.886612	0.800181	hg38_knownGene_ENST00000368130.9	1456	1464	+	tcccagaaa

Figure1

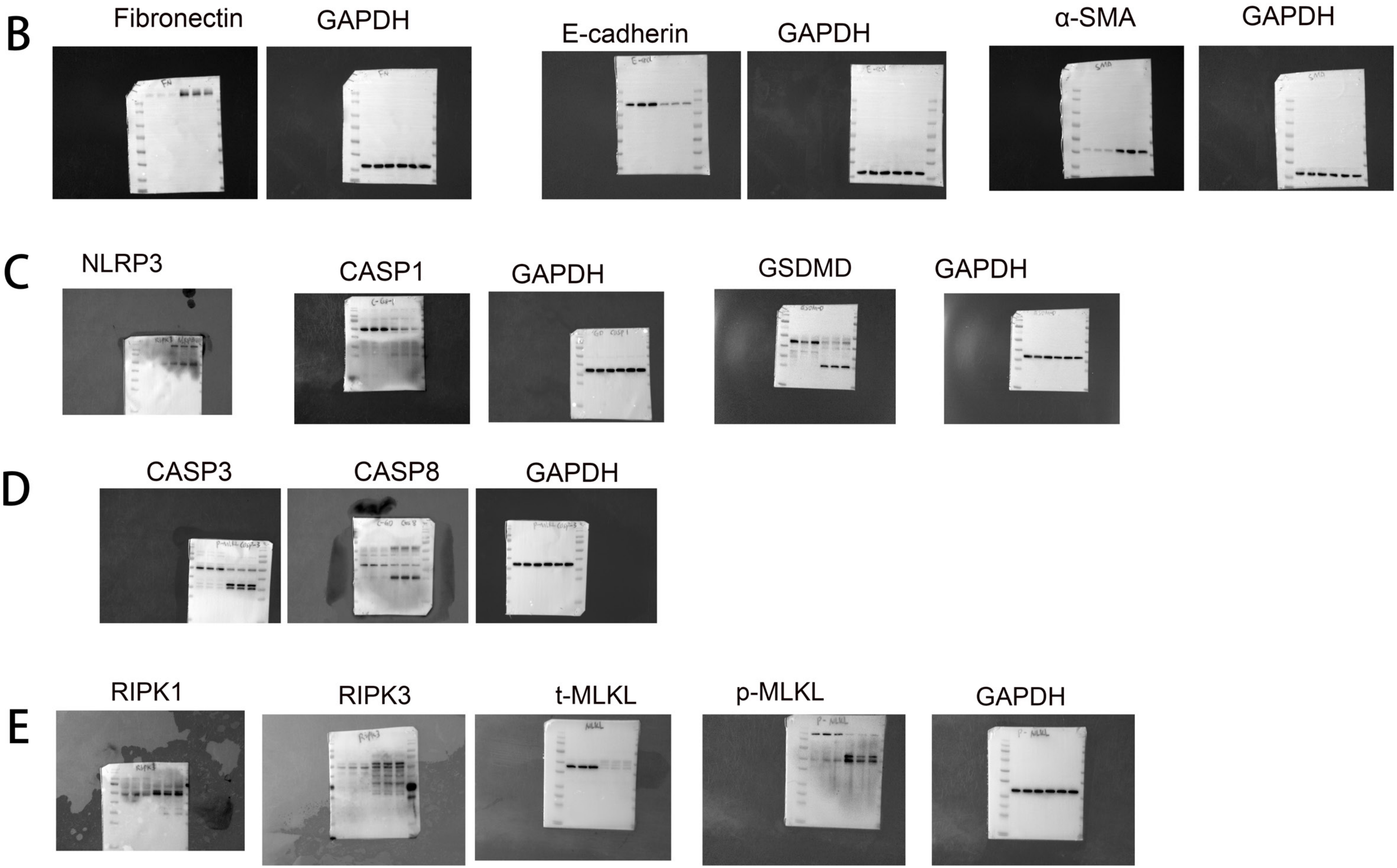
B
Fibronectin
GAPDH
E-cadherin
GAPDH
α-SMA
GAPDH
C
NLRP3
CASP1
GAPDH
GSDMD
GAPDH
D
CASP3
CASP8
GAPDH
E
RIPK1
RIPK3
t-MLKL
p-MLKL
GAPDH

Figure2

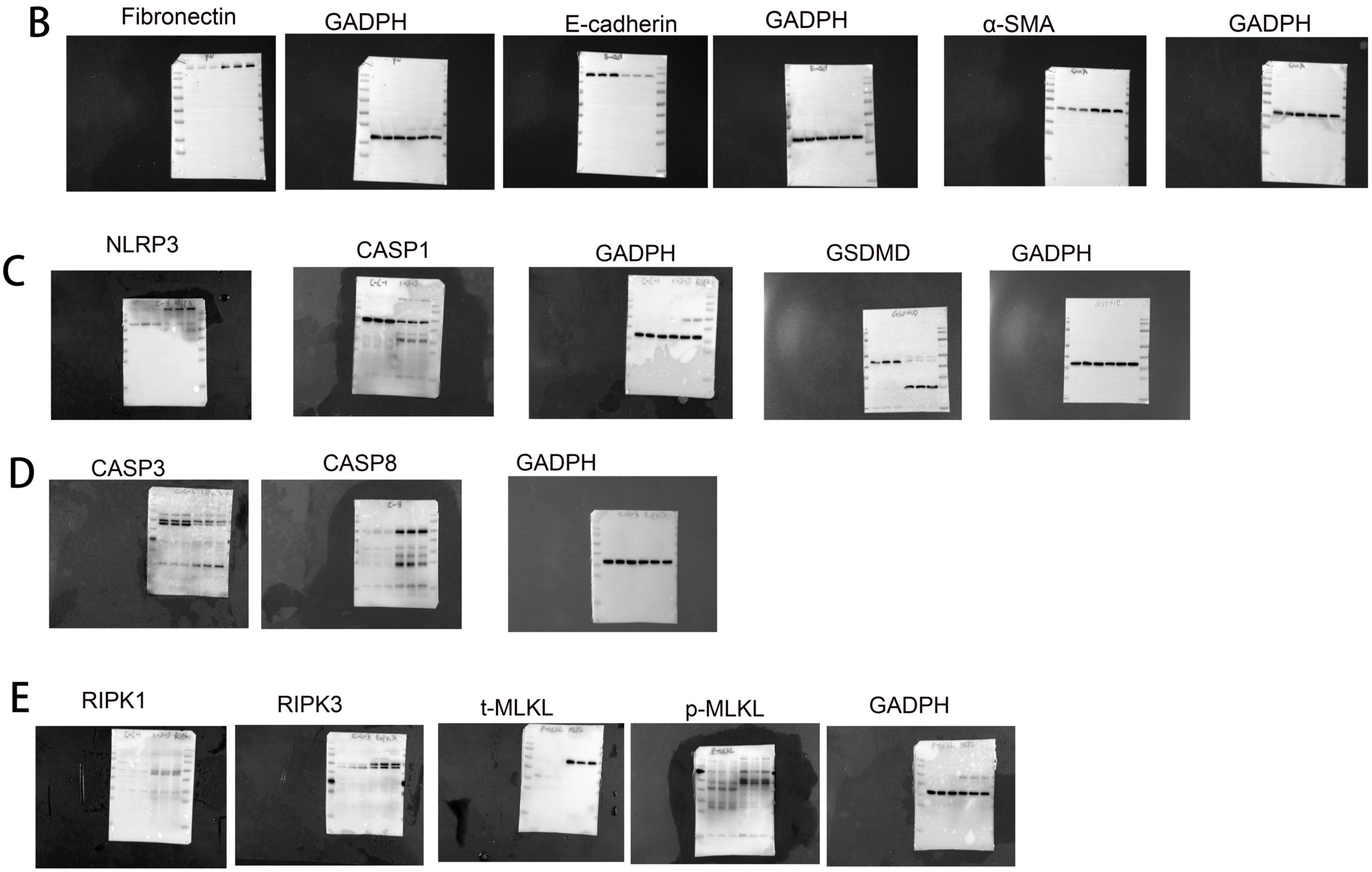
B
Fibronectin
GADPH
E-cadherin
GADPH
α-SMA
GADPH
C
NLRP3
CASP1
GADPH
GSDMD
GADPH
D
CASP3
CASP8
GADPH
E
RIPK1
RIPK3
t-MLKL
p-MLKL
GADPH

Figure4

C

TNF-α GAPDH

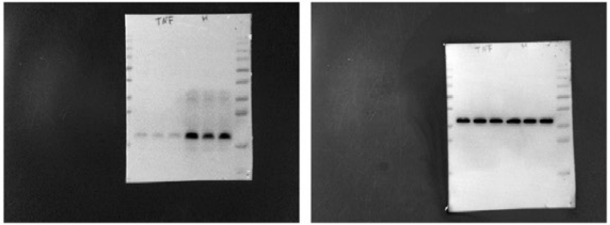

F

TNF-α GAPDH

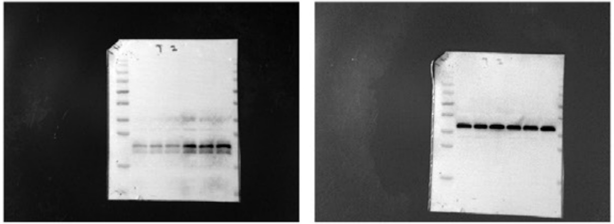

Figure5

E

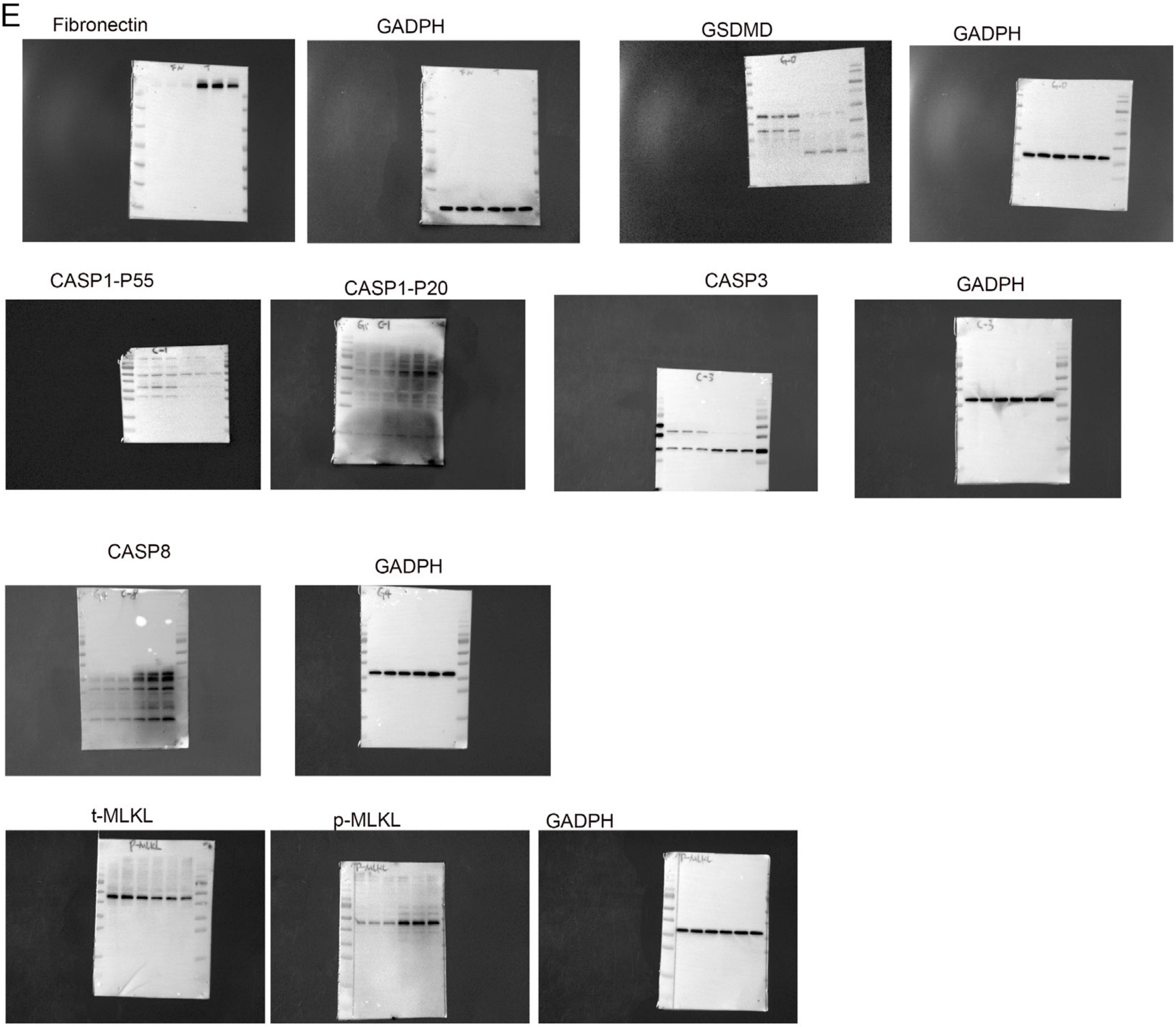
Fibronectin
GADPH
GSDMD
GADPH
CASP1-P55
CASP1-P20
CASP3
GADPH
CASP8
GADPH
t-MLKL
p-MLKL
GADPH

I

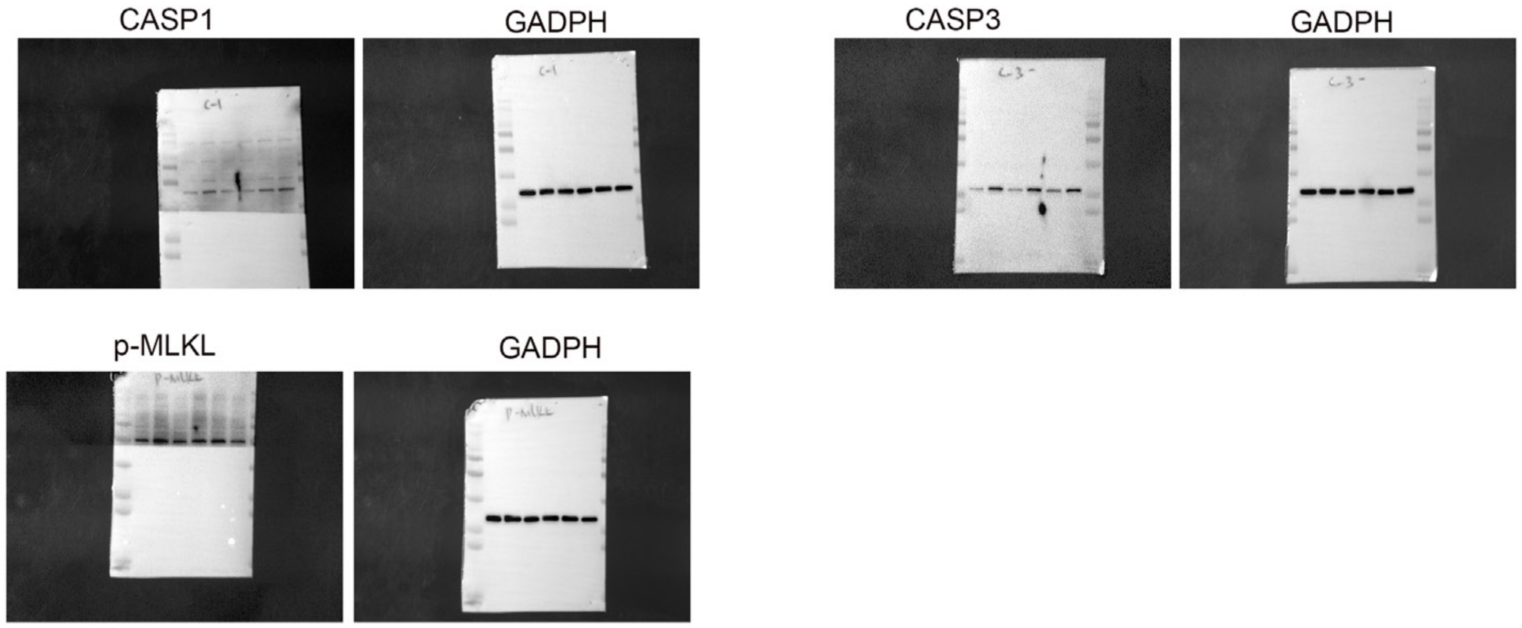
CASP1
GADPH
CASP3
GADPH
p-MLKL
GADPH

Figure6

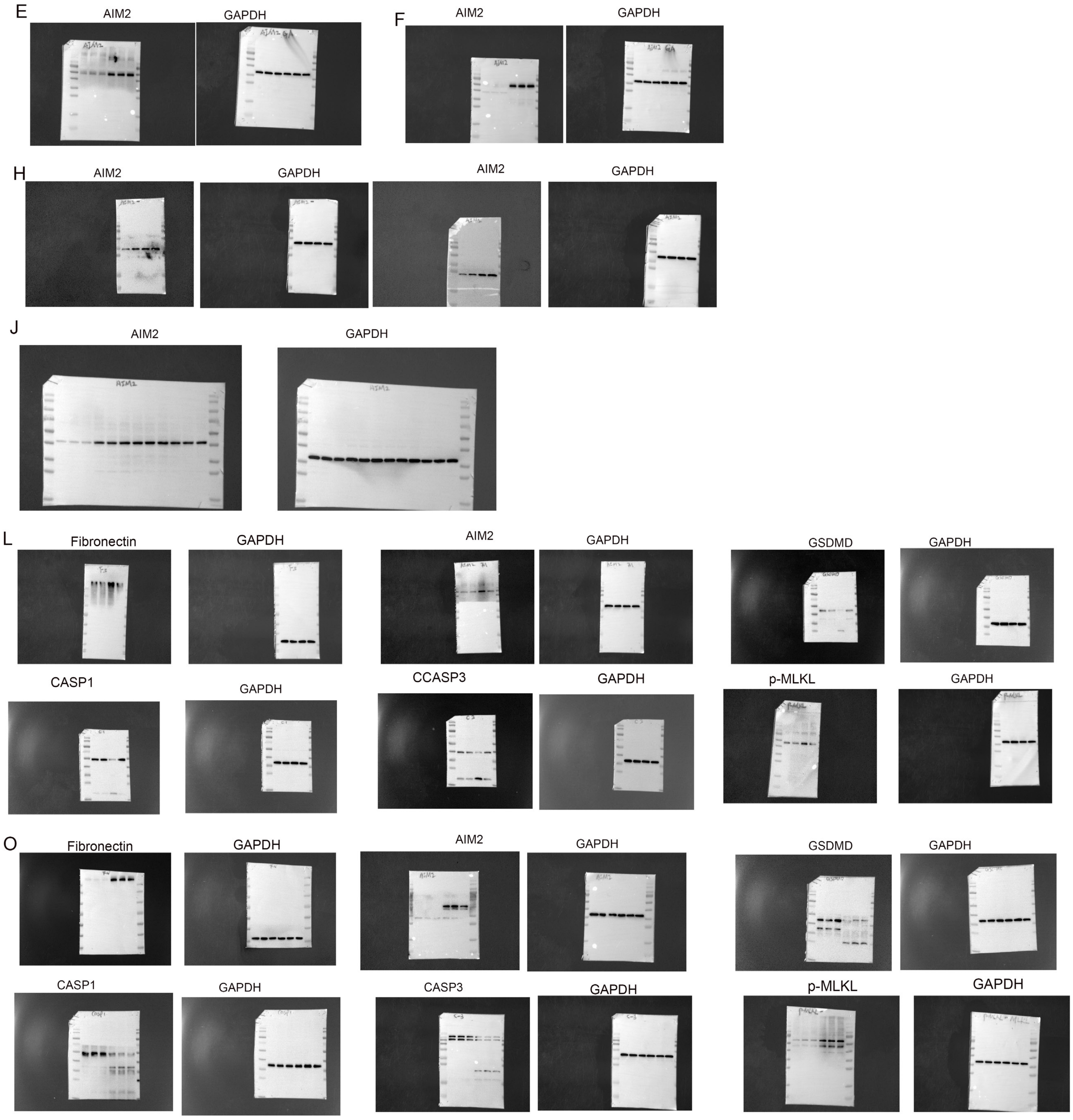
E
AIM2
GAPDH
F
AIM2
GAPDH
H
AIM2
GAPDH
AIM2
GAPDH
J
AIM2
GAPDH
L
Fibronectin
GAPDH
AIM2
GAPDH
GSDMD
GAPDH
CASP1
GAPDH
CCASP3
GAPDH
p-MLKL
GAPDH
O
Fibronectin
GAPDH
AIM2
GAPDH
GSDMD
GAPDH
CASP1
GAPDH
CASP3
GAPDH
p-MLKL
GAPDH

Figure7

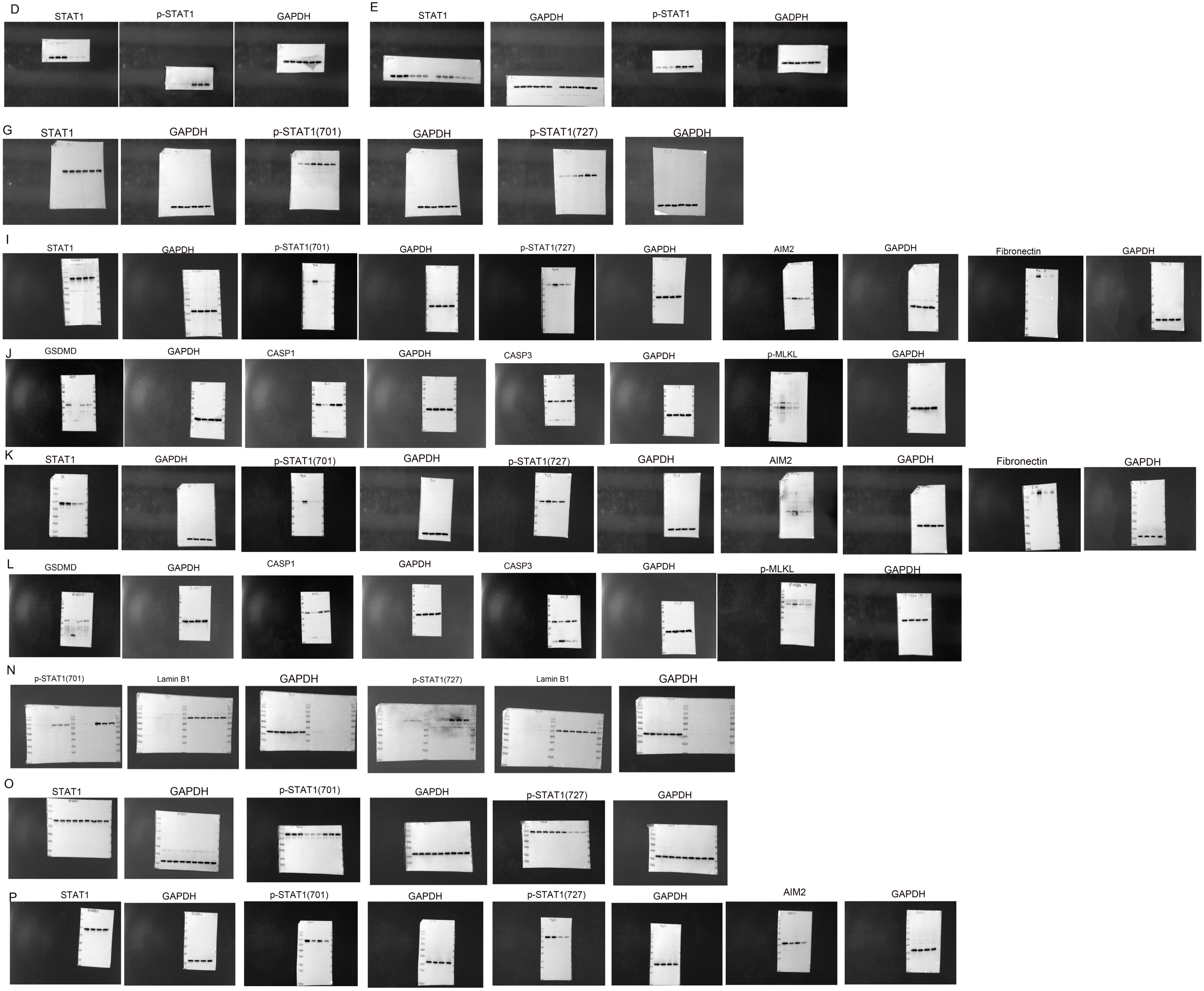
D
STAT1
p-STAT1
GAPDH
E
STAT1
GAPDH
p-STAT1
GADPH
G
STAT1
GAPDH
p-STAT1(701)
GAPDH
p-STAT1(727)
GAPDH
I
STAT1
GAPDH
p-STAT1(701)
GAPDH
p-STAT1(727)
GAPDH
AIM2
GAPDH
Fibronectin
GAPDH
J
GSDMD
GAPDH
CASP1
GAPDH
CASP3
GAPDH
p-MLKL
GAPDH
K
STAT1
GAPDH
p-STAT1(701)
GAPDH
p-STAT1(727)
GAPDH
AIM2
GAPDH
Fibronectin
GAPDH
L
GSDMD
GAPDH
CASP1
GAPDH
CASP3
GAPDH
p-MLKL
GAPDH
N
p-STAT1(701)
Lamin B1
GAPDH
p-STAT1(727)
Lamin B1
GAPDH
O
STAT1
GAPDH
p-STAT1(701)
GAPDH
p-STAT1(727)
GAPDH
P
STAT1
GAPDH
p-STAT1(701)
GAPDH
p-STAT1(727)
GAPDH
AIM2
GAPDH

Figure8

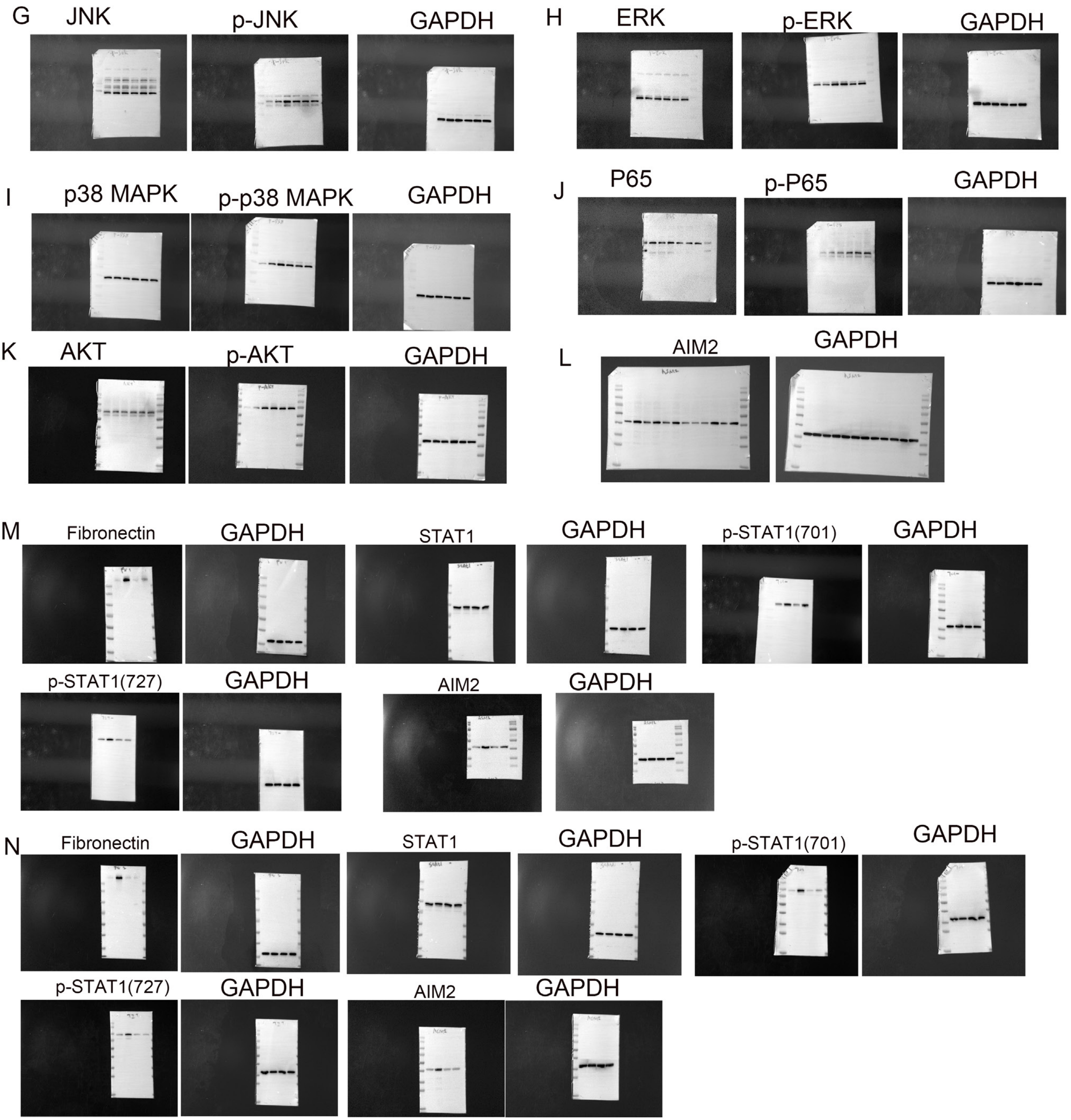
G
JNK
p-JNK
GAPDH
H
ERK
p-ERK
GAPDH
I
p38 MAPK
p-p38 MAPK
GAPDH
J
P65
p-P65
GAPDH
K
AKT
p-AKT
GAPDH
L
AIM2
GAPDH
M
Fibronectin
GAPDH
STAT1
GAPDH
p-STAT1(701)
GAPDH
p-STAT1(727)
GAPDH
AIM2
GAPDH
N
Fibronectin
GAPDH
STAT1
GAPDH
p-STAT1(701)
GAPDH
p-STAT1(727)
GAPDH
AIM2
GAPDH

Figure9

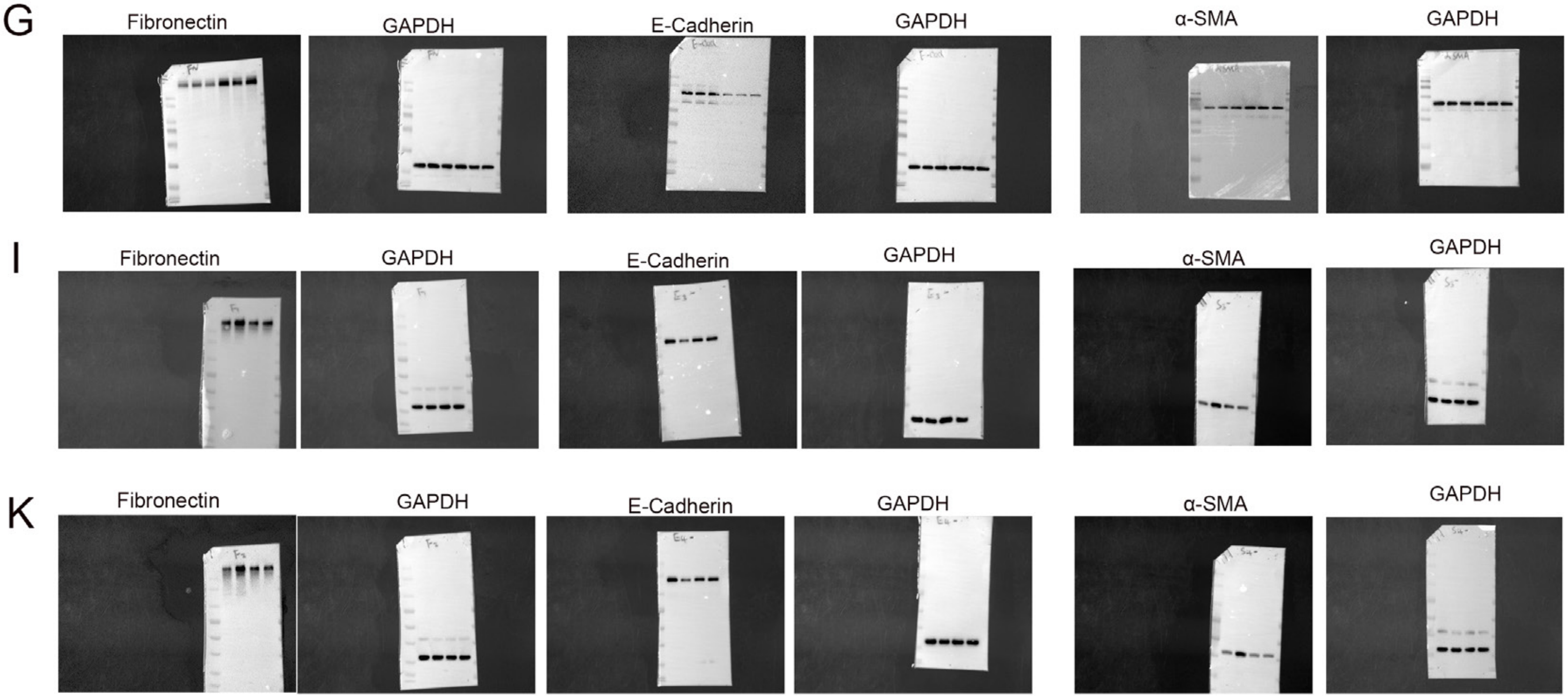
G
Fibronectin
GAPDH
E-Cadherin
GAPDH
α-SMA
GAPDH
I
Fibronectin
GAPDH
E-Cadherin
GAPDH
α-SMA
GAPDH
K
Fibronectin
GAPDH
E-Cadherin
GAPDH
α-SMA
GAPDH

Figure S5

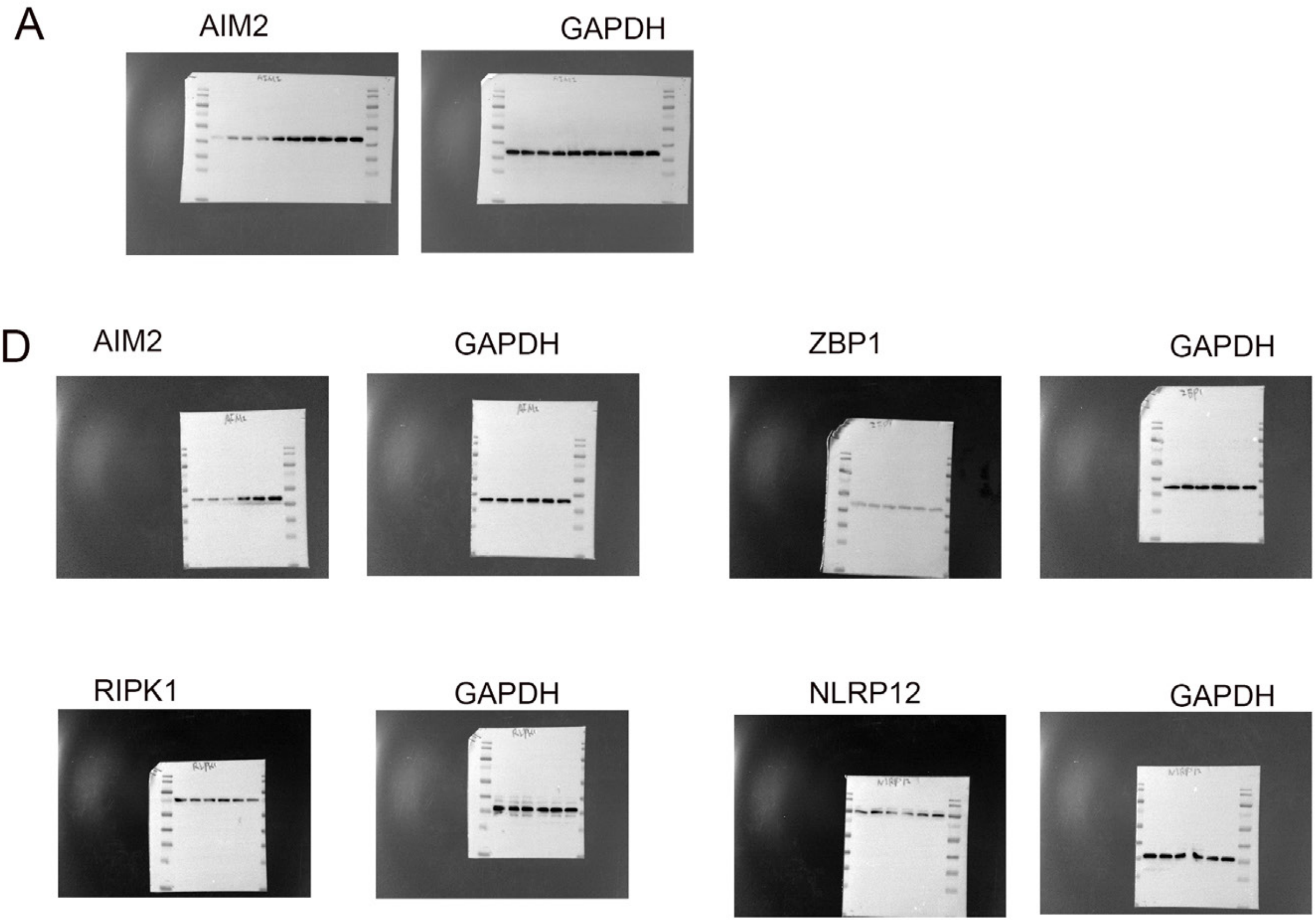

A
AIM2
GAPDH
D
AIM2
GAPDH
ZBP1
GAPDH
RIPK1
GAPDH
NLRP12
GAPDH

Figure S6

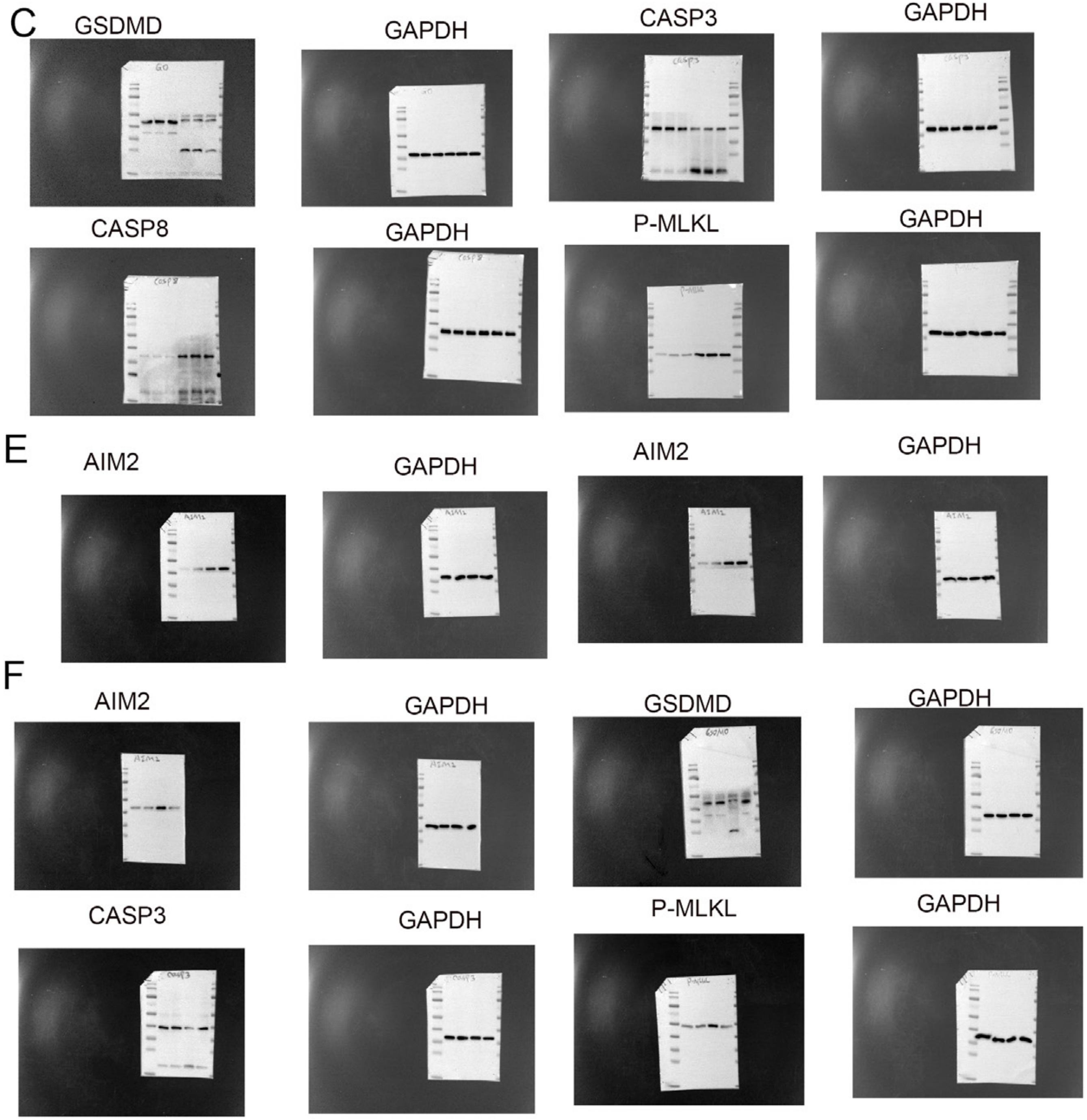
C
GSDMD
GAPDH
CASP3
GAPDH
CASP8
GAPDH
P-MLKL
GAPDH
E
AIM2
GAPDH
AIM2
GAPDH
F
AIM2
GAPDH
GSDMD
GAPDH
CASP3
GAPDH
P-MLKL
GAPDH

Figure S9

B

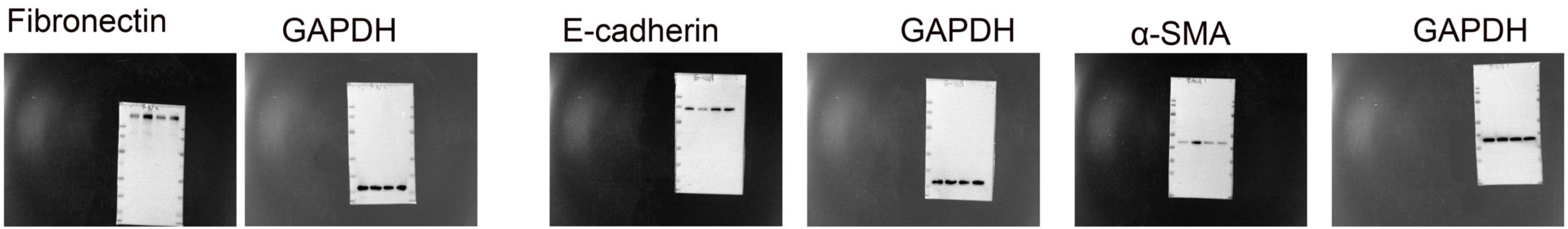
Fibronectin
GAPDH
E-cadherin
GAPDH
α-SMA
GAPDH

D

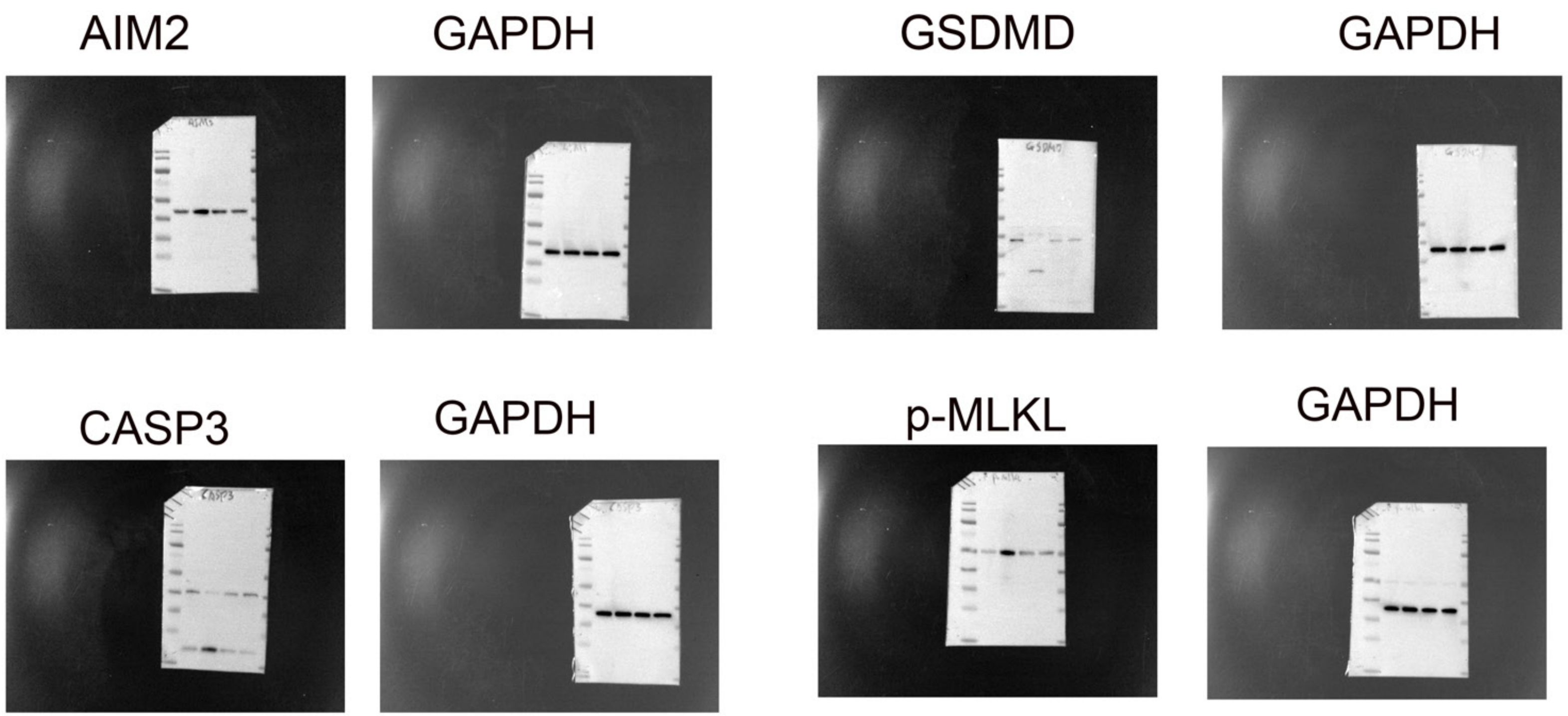
AIM2
GAPDH
GSDMD
GAPDH
CASP3
GAPDH
p-MLKL
GAPDH

Figure S10

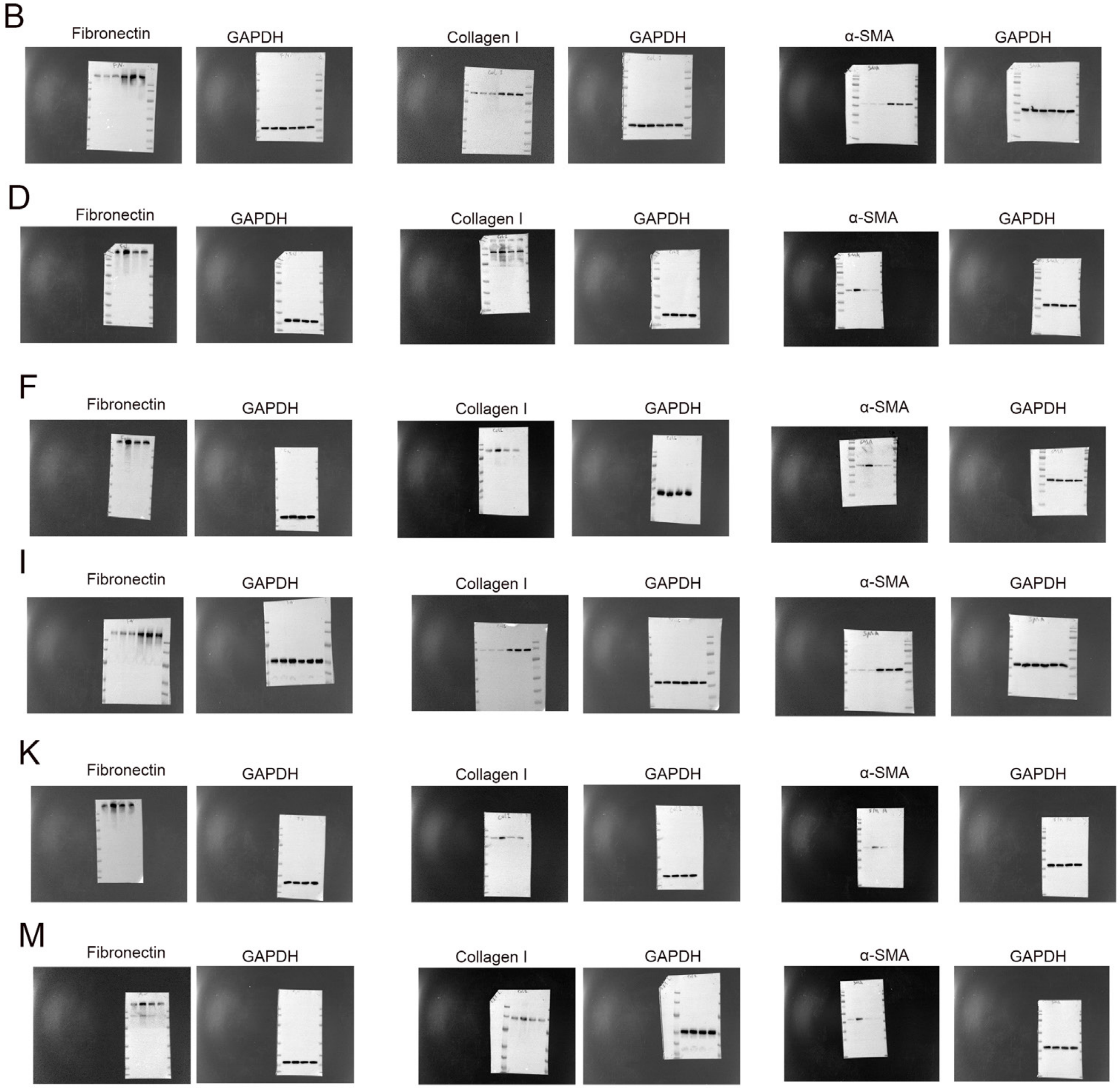
B
Fibronectin
GAPDH
Collagen I
GAPDH
α-SMA
GAPDH
D
Fibronectin
GAPDH
Collagen I
GAPDH
α-SMA
GAPDH
F
Fibronectin
GAPDH
Collagen I
GAPDH
α-SMA
GAPDH
I
Fibronectin
GAPDH
Collagen I
GAPDH
α-SMA
GAPDH
K
Fibronectin
GAPDH
Collagen I
GAPDH
α-SMA
GAPDH
M
Fibronectin
GAPDH
Collagen I
GAPDH
α-SMA
GAPDH
